# Supplementary material for: Interventions to reduce stigma related to contraception and abortion: a scoping review
Source: BMJ Open. 2022 Nov 16;12(11):e063870. doi: 10.1136/bmjopen-2022-063870 (PMC9677031; doi:10.1136/bmjopen-2022-063870)
Supplement: Supplementary data [file bmjopen-2022-063870supp002.pdf]

APPENDICES: DATABASE SEARCH STRATEGIES

Scoping review of interventions to reduce stigma related to contraception and abortion
Medline
2 Feb 2022 – Update conducted
Limited to 2000-Current

	#	Searches	Results
**Stigma**		Social stigma* OR shame* OR guilt* OR discriminat* OR Prejudice* OR judgement OR stereotype*OR blame OR isolate* OR moral* OR harass* OR insult* OR labeling OR social-accept* OR social-approv* OR social-percep*OR treatment-barrier*	101353
**Abortion or contraception**		abortion, induced OR Abortion, Criminal OR Abortion, Septic OR Abortion Applicants OR abortion, missed OR abortion, legal OR abortion, spontaneous OR abortion* OR pregnancy-terminat* OR post-abortion*OR Postconception Fertility Control OR Embryotomies OR Embryotomy OR Dilatation and curettage OR Vacuum Curettage OR Surgical abortion OR Manual vacuum aspiration OR (vacuum AND aspiration) OR Curettage OR Surgical termination of pregnancy OR Dilatation and evacuation OR Dilation and evacuation OR Suction aspiration OR Aspiration abortion OR Suction curettage OR Vacuum curettage OR Contraception OR Contraception Behavior OR Condom OR Condoms OR Diaphragm OR Diaphragms OR Cervical Cap OR Vaginal-sponge OR Cervical-Caps OR Vaginal-sponges OR Birth-Control OR Contraceptive-Methods OR Contraceptive-Method OR Female Contraception OR Female Contraceptions OR Male Contraception OR Male Contraceptions OR Inhibition-of-Fertilization OR Fertilization-Inhibition OR Fertility-Control OR Birth-Control OR Vaginal-Barrier OR Hormonal-Contracept* OR anticonception OR antifertility OR conception-control OR family planning OR birth control OR birth-prevention OR pregnancy-prevent*	5257

**Programe valuation-related**		intervention OR program OR activity OR action	307914
		1+2+3	41 (only 10 non-duplicates)

Scoping review of interventions to reduce stigma related to contraception and abortion
PUBMED
Updated – 2 February 2022
Limited to 2021/10/01 to present

	#	Searches	Results
**Stigma**		Social Stigma[Mesh] OR Social stigma*[Tiab] OR shame*[tiab] OR guilt*[tiab] OR discriminat*[tiab] OR Prejudice*[tiab] OR judgement[tiab] OR stereotype*[tiab] OR blame[tiab] OR isolate*[tiab] OR moral*[tiab] OR harass*[tiab] OR insult*[tiab] OR labeling[tiab] OR social-accept*[tiab] OR social-approv*[tiab] OR social-percep*[tiab] OR treatment-barrier*[tiab]	
**Abortion and contraception**		abortion, induced[MeSH Terms] OR Abortion, Criminal[Mesh] OR Abortion, Septic[Mesh] OR Abortion Applicants[Mesh] OR abortion, missed[MeSH Terms] OR "abortion, legal"[MeSH Terms] OR abortion, spontaneous[MeSH Terms] OR abortion*[Tiab] OR pregnancy-terminat*[tiab] OR post-abortion*[tiab] OR (pregnan*[TW] AND terminat*[TW]) OR (Abort*[TW] AND Pregnan*[TW]) OR Postconception Fertility Control[TIAB] OR Embryotomies[TIAB] OR Embryotomy[TIAB] OR Dilatation and curettage[MeSH] OR Vacuum Curettage[MeSH] OR Surgical abortion[tiab] OR Manual vacuum aspiration[tiab] OR (vacuum[tiab] AND aspiration[tiab]) OR Curettage[tiab] OR Surgical termination of pregnancy[tiab] OR Dilatation and evacuation[tiab] OR Dilation and evacuation[tiab] OR Suction aspiration[tiab] OR Aspiration abortion[tiab] OR Suction curettage[tiab] OR Vacuum curettage[tiab] OR Contraception[Mesh] OR Contraception Behavior[Mesh] OR Condom[TIAB] OR Condoms[TIAB] OR Diaphragm[TIAB] OR Diaphragms [TIAB] OR Cervical Cap [TIAB] OR Vaginal-sponge[tiab] OR Cervical-Caps [TIAB] OR Vaginal-sponges OR Birth-Control [TIAB] OR Contraceptive-Methods [TIAB] OR Contraceptive-Method [TIAB] OR Female Contraception [TIAB] OR Female Contraceptions [TIAB] OR Male Contraception [TIAB] OR Male Contraceptions [TIAB] OR Inhibition-of-Fertilization[tiab] OR Fertilization-Inhibition[tiab] OR Fertility-Control[tiab] OR Birth-Control[tiab] OR Vaginal-Barrier[tiab] OR Hormonal-Contracept*[tiab] OR anticonception[tiab] OR antifertility[tiab] OR conception-control[tiab] OR family planning[TIAB] OR birth control [tiab] OR birth-prevention[tiab] OR pregnancy-prevent*[tiab]	
**Intervention**		intervention[mesh] OR intervention*[TIAB] OR activity[mesh] OR activity [TIAB]	

**TOTAL**			8 after duplicates removed

Scoping review of interventions to reduce stigma related to contraception and abortion
EMBASE
19 October 2021 – Search conducted
Limited to 01/01/2001-Current

	#	**Searches**	**Results**
**Stigma**		Social stigma* OR shame* OR guilt* OR discriminat* OR Prejudice* OR judgement OR stereotype*OR blame OR isolate* OR moral* OR harass* OR insult* OR labeling OR social-accept* OR social-approv* OR social-percep*OR treatment-barrier*	116281
**Abortion or contraception**		abortion, induced OR Abortion, Criminal OR Abortion, Septic OR Abortion Applicants OR abortion, missed OR abortion, legal OR abortion, spontaneous OR abortion* OR pregnancy-terminat* OR post-abortion*OR Postconception Fertility Control OR Embryotomies OR Embryotomy OR Dilatation and curettage OR Vacuum Curettage OR Surgical abortion OR Manual vacuum aspiration OR (vacuum AND aspiration) OR Curettage OR Surgical termination of pregnancy OR Dilatation and evacuation OR Dilation and evacuation OR Suction aspiration OR Aspiration abortion OR Suction curettage OR Vacuum curettage OR Contraception OR Contraception Behavior OR Condom OR Condoms OR Diaphragm OR Diaphragms OR Cervical Cap OR Vaginal-sponge OR Cervical-Caps OR Vaginal-sponges OR Birth-Control OR Contraceptive-Methods OR Contraceptive-Method OR Female Contraception OR Female Contraceptions OR Male Contraception OR Male Contraceptions OR Inhibition-of-Fertilization OR Fertilization-Inhibition OR Fertility-Control OR Birth-Control OR Vaginal-Barrier OR Hormonal-Contracept* OR anticonception OR antifertility OR conception-control OR family planning OR birth control OR birth-prevention OR pregnancy-prevent*	8283

**Programevaluation-related**		intervention OR program OR activity OR action	417836
		1+2+3	104

Scoping review of interventions to reduce stigma related to contraception and abortion
Web of Science 19 October 2021 – Search conducted
Limited to 2000-01-01 to 2021-12-01

	#	Searches	Results
**Stigma**		Social stigma* OR shame* OR guilt* OR discriminat* OR Prejudice* OR judgement OR stereotype*OR blame OR isolate* OR moral* OR harass* OR insult* OR labeling OR social-accept* OR social-approv* OR social-percep*OR treatment-barrier*	
**Abortion or contraception**		abortion, induced OR Abortion, Criminal OR Abortion, Septic OR Abortion Applicants OR abortion, missed OR abortion, legal OR abortion, spontaneous OR abortion* OR pregnancy-terminat* OR post-abortion*OR Postconception Fertility Control OR Embryotomies OR Embryotomy OR Dilatation and curettage OR Vacuum Curettage OR Surgical abortion OR Manual vacuum aspiration OR (vacuum AND aspiration) OR Curettage OR Surgical termination of pregnancy OR Dilatation and evacuation OR Dilation and evacuation OR Suction aspiration OR Aspiration abortion OR Suction curettage OR Vacuum curettage OR Contraception OR Contraception Behavior OR Condom OR Condoms OR Diaphragm OR Diaphragms OR Cervical Cap OR Vaginal-sponge OR Cervical-Caps OR Vaginal-sponges OR Birth-Control OR Contraceptive-Methods OR Contraceptive-Method OR Female Contraception OR Female Contraceptions OR Male Contraception OR Male Contraceptions OR Inhibition-of-Fertilization OR Fertilization-Inhibition OR Fertility-Control OR Birth-Control OR Vaginal-Barrier OR Hormonal-Contracept* OR anticonception OR antifertility OR conception-control OR family planning OR birth control OR birth-prevention OR pregnancy-prevent*	

**Programevaluation-related**		intervention OR program OR activity OR action	
		1+2+3	1014<br>1827<br>4840

Scoping review of interventions to reduce stigma related to contraception and abortion
PsycInfo - updated 2 February 2022
Limited to 2000-Current

	#	Searches	Results
**Stigma**		Social stigma* OR shame* OR guilt* OR discriminat* OR Prejudice* OR judgement OR stereotype*OR blame OR isolate* OR moral* OR harass* OR insult* OR labeling OR social-accept* OR social-approv* OR social-percep*OR treatment-barrier*	11397
**Abortion or contraception**		abortion, induced OR Abortion, Criminal OR Abortion, Septic OR Abortion Applicants OR abortion, missed OR abortion, legal OR abortion, spontaneous OR abortion* OR pregnancy-terminat* OR post-abortion*OR Postconception Fertility Control OR Embryotomies OR Embryotomy OR Dilatation and curettage OR Vacuum Curettage OR Surgical abortion OR Manual vacuum aspiration OR (vacuum AND aspiration) OR Curettage OR Surgical termination of pregnancy OR Dilatation and evacuation OR Dilation and evacuation OR Suction aspiration OR Aspiration abortion OR Suction curettage OR Vacuum curettage OR Contraception OR Contraception Behavior OR Condom OR Condoms OR Diaphragm OR Diaphragms OR Cervical Cap OR Vaginal-sponge OR Cervical-Caps OR Vaginal-sponges OR Birth-Control OR Contraceptive-Methods OR Contraceptive-Method OR Female Contraception OR Female Contraceptions OR Male Contraception OR Male Contraceptions OR Inhibition-of-Fertilization OR Fertilization-Inhibition OR Fertility-Control OR Birth-Control OR Vaginal-Barrier OR Hormonal-Contracept* OR anticonception OR antifertility OR conception-control OR family planning OR birth control OR birth-prevention OR pregnancy-prevent*	530

**Programevaluation-related**		intervention OR program OR activity OR action	36689
		1+2+3	8 (2 non-duplicates)
