# Supplementary material for: Interventions to reduce stigma related to contraception and abortion: a scoping review
Source: BMJ Open. 2022 Nov 16;12(11):e063870. doi: 10.1136/bmjopen-2022-063870 (PMC9677031; doi:10.1136/bmjopen-2022-063870)
Supplement: Supplementary data [file bmjopen-2022-063870supp003.pdf]

Supplemental material

Table 2. Characteristics of included studies (n=18)

Author	Year of publication	Country	Scope	Evaluation design	Focus	Type of stigma	Intervention approach
Banerjee	2013	India	To evaluate the effectiveness of a behavior change communication intervention to improve women's knowledge about India's abortion law and their perceptions about abortion	Quasi-experimental	Abortion	Community	Education/training/skills building
Belfarge	2020	Mexico	The evaluation measured whether participation in the intervention reduced women's experience of individual level stigma one month after having participated and explored women's expressed experiences of the intervention and their perceptions of changes in stigmatising feelings after the intervention.	Mixed methods	Abortion	Intrapersonal	Counselling/peer support
Belfarge	2021	Mexico	To understand more about who tells their stories online, how they represent their abortion experience, and what lessons this leaves us as feminist activists seeking to advance sexual and reproductive rights through online storytelling	Qualitative	Abortion	Intrapersonal	Contact
Cockrill	2018	USA	To measure changes in women's attitudes toward abortion and their willingness to disclose their own abortion experiences with one another	Quasi-experimental	Abortion	Interpersonal	Contact

			following a book group discussion in which participants discussed a previously chosen book.				
Guiahi	2011	USA	To evaluate if a values clarification workshop conducted at Catholic hospital training programs influenced obstetrics and gynecology residents' abortion attitudes.	Quasi-experi mental	Abortion	Interpersonal	Education/training/skills building
Guiahi	2021	USA	To understand whether a values clarification workshop influences abortion attitudes of obgyn residents at U.S. training programs that are based in Catholic hospitals and lack abortion training.	Quasi-experi mental	Abortion	Interpersonal	Education/training/skills building
Harris	2011	USA	To present findings from a qualitative pilot study of the Providers Share Workshop, a six-session workshop in which abortion providers (broadly defined to include anyone with direct daily involvement in abortion care), meet to explore their experiences, guided by an experienced facilitator	Qualitative	Abortion	Intrapersonal	Counselling/peer support
Keefe-Oates	2020	Colombia and Mexico City	To develop a greater understanding of their clients' concerns prior to abortion care,and the role that counselling can play in addressing these concerns.	Qualitative	Abortion	Intrapersonal	Counselling/peer support
Martin	2014	USA	The Providers Share Workshop (PSW) provides abortion providers safe space to discuss	Quasi-experi mental	Abortion	Intrapersonal	Counselling/peer support

			their work experiences. Our objectives were to assess changes in abortion stigma over time and explore how stigma is related to aspects of professional quality of life, including compassion satisfaction, burnout and compassion fatigue for providers participating in the workshops.				
Mosley	2020	Sub-Saharan Africa and Latin American and the Caribbean*	To adapt the Providers Share Workshop content, structure and evaluation tools for this pilot study in Sub-Saharan Africa and Latin America.	Cohort	Abortion	Intrapersonal	Counselling/peer support
Mpeli	2015	South Africa	To report on the value clarification process and the views of preregistration midwifery students regarding abortion-related services before and after implementing the value clarification process.	Qualitative	Abortion	Intrapersonal	Education/training/skills building
Palinggi	2021	Indonesia	To analyze the influence of the Balanced Counseling Strategy (FP-BCS) on attitudes, subjective norms, and intentions on modern contraception in the working area of Singgani Health Center, Palu, Indonesia	Quasi-experimental	Contraception	Community	Education/training/skills building
Rehnström Loi	2020	Kenya	To measure attitudes towards abortion and contraceptive use, and to evaluate a stigma-reduction intervention	Quasi-experimental	Both	Interpersonal	Education/training/skills building

			among secondary school students.				
Sackeim	2022	USA	To evaluate the effect of a narrative intervention on individual-level abortion stigma in patients undergoing abortion.	Randomized Controlled Trial	Abortion	Intrapersonal	Contact
San-Martos	2021	Spain	This study aimed to evaluate the effect of a peer-educational intervention to improve knowledge level and attitudes regarding contraceptive methods in university students.	Quasi-experi mental	Contraceptio n	Community	Education/training/skills building
Sisson	2016	USA	To determine whether a documentary film can impact stigmatizing attaitudes against 3rd trimester abortion.	Qualitative	Abortion	Community	Media
Sisson	2021	USA	To examine the impact of an abortion storyline from the TV show Grey's Anatomy on USA-based viewers.	Quasi-experi mental	Abortion	Community	Media
Smith	2021	Cambodia	The aim of this study was to describe the development of an intervention to support the reproductive health of garment factory workers in Cambodia	Qualitative	Both	Community	Media

*Only regions were report

Appendix 1. Preferred Reporting Items for Systematic reviews and Meta-Analyses extension for Scoping Reviews (PRISMA-ScR) Checklist

SECTION	ITEM	PRISMA-ScR CHECKLIST ITEM	REPORTED ON PAGE #
**TITLE**			
Title	1	Identify the report as a scoping review.	1
**ABSTRACT**			
Structured summary	2	Provide a structured summary that includes (as applicable): background, objectives, eligibility criteria, sources of evidence, charting methods, results, and conclusions that relate to the review questions and objectives.	2
**INTRODUCTION**			
Rationale	3	Describe the rationale for the review in the context of what is already known. Explain why the review questions/objectives lend themselves to a scoping review approach.	4
Objectives	4	Provide an explicit statement of the questions and objectives being addressed with reference to their key elements (e.g., population or participants, concepts, and context) or other relevant key elements used to conceptualize the review questions and/or objectives.	4
**METHODS**			
Protocol and registration	5	Indicate whether a review protocol exists; state if and where it can be accessed (e.g., a Web address); and if available, provide registration information, including the registration number.	N/A
Eligibility criteria	6	Specify characteristics of the sources of evidence used as eligibility criteria (e.g., years considered, language, and publication status), and provide a rationale.	5
Information sources*	7	Describe all information sources in the search (e.g., databases with dates of coverage and contact with authors to identify additional sources), as well as the date the most recent search was executed.	5
Search	8	Present the full electronic search strategy for at least 1 database, including any limits used, such that it could be repeated.	26
Selection of sources of evidence†	9	State the process for selecting sources of evidence (i.e., screening and eligibility) included in the scoping review.	5
Data charting process‡	10	Describe the methods of charting data from the included sources of evidence (e.g., calibrated forms or forms that have been tested by the team before their use, and whether data charting was done independently	5-6

SECTION	ITEM	PRISMA-ScR CHECKLIST ITEM	REPORTED ON PAGE #
		or in duplicate) and any processes for obtaining and confirming data from investigators.	
Data items	11	List and define all variables for which data were sought and any assumptions and simplifications made.	22
Critical appraisal of individual sources of evidence§	12	If done, provide a rationale for conducting a critical appraisal of included sources of evidence; describe the methods used and how this information was used in any data synthesis (if appropriate).	N/A
Synthesis of results	13	Describe the methods of handling and summarizing the data that were charted.	6
**RESULTS**			
Selection of sources of evidence	14	Give numbers of sources of evidence screened, assessed for eligibility, and included in the review, with reasons for exclusions at each stage, ideally using a flow diagram.	15
Characteristics of sources of evidence	15	For each source of evidence, present characteristics for which data were charted and provide the citations.	16-21
Critical appraisal within sources of evidence	16	If done, present data on critical appraisal of included sources of evidence (see item 12).	N/A
Results of individual sources of evidence	17	For each included source of evidence, present the relevant data that were charted that relate to the review questions and objectives.	Click here to enter text.
Synthesis of results	18	Summarize and/or present the charting results as they relate to the review questions and objectives.	7-9
**DISCUSSION**			
Summary of evidence	19	Summarize the main results (including an overview of concepts, themes, and types of evidence available), link to the review questions and objectives, and consider the relevance to key groups.	9-10
Limitations	20	Discuss the limitations of the scoping review process.	10
Conclusions	21	Provide a general interpretation of the results with respect to the review questions and objectives, as well as potential implications and/or next steps.	9-10
**FUNDING**			
Funding	22	Describe sources of funding for the included sources of evidence, as well as sources of funding for the scoping review. Describe the role of the funders of the scoping review.	10

JBI = Joanna Briggs Institute; PRISMA-ScR = Preferred Reporting Items for Systematic reviews and Meta-Analyses extension for Scoping Reviews.
* Where *sources of evidence* (see second footnote) are compiled from, such as bibliographic databases, social media platforms, and Web sites.

† A more inclusive/heterogeneous term used to account for the different types of evidence or data sources (e.g., quantitative and/or qualitative research, expert opinion, and policy documents) that may be eligible in a scoping review as opposed to only studies. This is not to be confused with *information sources* (see first footnote).
‡ The frameworks by Arksey and O'Malley (6) and Levac and colleagues (7) and the JBI guidance (4, 5) refer to the process of data extraction in a scoping review as data charting.
§ The process of systematically examining research evidence to assess its validity, results, and relevance before using it to inform a decision. This term is used for items 12 and 19 instead of "risk of bias" (which is more applicable to systematic reviews of interventions) to include and acknowledge the various sources of evidence that may be used in a scoping review (e.g., quantitative and/or qualitative research, expert opinion, and policy document).

From: Tricco AC, Lillie E, Zarin W, O'Brien KK, Colquhoun H, Levac D, et al. PRISMA Extension for Scoping Reviews (PRISMAScR): Checklist and Explanation. Ann Intern Med. 2018;169:467–473. doi: 10.7326/M18-0850.

Appendix 2. Example search strategy

	#	**Searches**
**Stigma**		Social stigma* OR shame* OR guilt* OR discriminat* OR Prejudice* OR judgement OR stereotype*OR blame OR isolate* OR moral* OR harass* OR insult* OR labeling OR social-accept* OR social-approv* OR social-percep*OR treatment-barrier*
**Abortion or contraception**		abortion, induced OR Abortion, Criminal OR Abortion, Septic OR Abortion Applicants OR abortion, missed OR abortion, legal OR abortion, spontaneous OR abortion* OR pregnancy-terminat* OR post-abortion*OR Postconception Fertility Control OR Embryotomies OR Embryotomy OR Dilatation and curettage OR Vacuum Curettage OR Surgical abortion OR Manual vacuum aspiration OR (vacuum AND aspiration) OR Curettage OR Surgical termination of pregnancy OR Dilatation and evacuation OR Dilation and evacuation OR Suction aspiration OR Aspiration abortion OR Suction curettage OR Vacuum curettage OR Contraception OR Contraception Behavior OR Condom OR Condoms OR Diaphragm OR Diaphragms OR Cervical Cap OR Vaginal-sponge OR Cervical-Caps OR Vaginal-sponges OR Birth-Control OR Contraceptive-Methods OR Contraceptive-Method OR Female Contraception OR Female Contraceptions OR Male Contraception OR Male Contraceptions OR Inhibition-of-Fertilization OR Fertilization-Inhibition OR Fertility-Control OR Birth-Control OR Vaginal-Barrier OR Hormonal-Contracept* OR anticonception OR antifertility OR conception-control OR family planning OR birth control OR birth-prevention OR pregnancy-prevent*

**Programevaluation-related**		intervention OR program OR activity OR action
