# Supplementary material for: Interventions to reduce stigma related to contraception and abortion: a scoping review
Source: BMJ Open. 2022 Nov 16;12(11):e063870. doi: 10.1136/bmjopen-2022-063870 (PMC9677031; doi:10.1136/bmjopen-2022-063870)
Supplement: Author's manuscript [file bmjopen-2022-063870.draft_revisions.pdf]

BMJ Open is committed to open peer review. As part of this commitment we make the peer review history of every article we publish publicly available.

When an article is published we post the peer reviewers' comments and the authors' responses online. We also post the versions of the paper that were used during peer review. These are the versions that the peer review comments apply to.

The versions of the paper that follow are the versions that were submitted during the peer review process. They are not the versions of record or the final published versions. They should not be cited or distributed as the published version of this manuscript.

BMJ Open is an open access journal and the full, final, typeset and author-corrected version of record of the manuscript is available on our site with no access controls, subscription charges or pay-per-view fees (http://bmjopen.bmj.com).

If you have any questions on BMJ Open's open peer review process please email info.bmjopen@bmj.com

BMJ Open

Interventions to reduce stigma related to contraception and abortion: a scoping review

Journal:	*BMJ Open*
Manuscript ID	bmjopen-2022-063870
Article Type:	Original research
Date Submitted by the Author:	28-Apr-2022
Complete List of Authors:	Sorhaindo, Annik; World Health Organization, Sexual and Reproductive Health and Research<br>Rehnstrom Loi, Ulrika; World Health Organization, Sexual and Reproductive Health and Research
Keywords:	PUBLIC HEALTH, REPRODUCTIVE MEDICINE, Reproductive medicine < GYNAECOLOGY

SCHOLARONE™
Manuscripts

I, the Submitting Author has the right to grant and does grant on behalf of all authors of the Work (as defined in the below author licence), an exclusive licence and/or a non-exclusive licence for contributions from authors who are: i) UK Crown employees; ii) where BMJ has agreed a CC-BY licence shall apply, and/or iii) in accordance with the terms applicable for US Federal Government officers or employees acting as part of their official duties; on a worldwide, perpetual, irrevocable, royalty-free basis to BMJ Publishing Group Ltd ("BMJ") its licensees and where the relevant Journal is co-owned by BMJ to the co-owners of the Journal, to publish the Work in this journal and any other BMJ products and to exploit all rights, as set out in our licence.

The Submitting Author accepts and understands that any supply made under these terms is made by BMJ to the Submitting Author unless you are acting as an employee on behalf of your employer or a postgraduate student of an affiliated institution which is paying any applicable article publishing charge ("APC") for Open Access articles. Where the Submitting Author wishes to make the Work available on an Open Access basis (and intends to pay the relevant APC), the terms of reuse of such Open Access shall be governed by a Creative Commons licence – details of these licences and which Creative Commons licence will apply to this Work are set out in our licence referred to above.

Other than as permitted in any relevant BMJ Author's Self Archiving Policies, I confirm this Work has not been accepted for publication elsewhere, is not being considered for publication elsewhere and does not duplicate material already published. I confirm all authors consent to publication of this Work and authorise the granting of this licence.

For peer review only

Title

Interventions to reduce stigma related to contraception and abortion: a scoping review

Authors

Annik Mahalia Sorhaindo, MSc PhD[1], Ulrika Rehnström Loi, MSc PhD[1*]

*Corresponding author: Ulrika Rehnström Loi, 20 Avenue Appia, 1211 Geneva, Switzerland, e-mail: rehnstromu@who.int

1. UNDP-UNFPA-UNICEF-WHO-World Bank Special Programme of Research, Development and Research Training in Human Reproduction (HRP), Department of Sexual and Reproductive Health and Research, World Health Organization, 20 Avenue Appia, 1211 Geneva, Switzerland

Key words (9): abortion stigma, contraceptive use stigma, values clarification, intervention, evaluation

For peer review only - http://bmjopen.bmj.com/site/about/guidelines.xhtml

Word count: 4659/5000 (including summary box, references, tables and figures)

Tables: 2 Figures: 1

For peer review only

For peer review only - http://bmjopen.bmj.com/site/about/guidelines.xhtml

Abstract

Objectives:

We conducted a scoping review to identify the types, volume and characteristics of available evidence and analyze gaps in the knowledge base for interventions to reduce contraception and abortion stigma.

Methods

We conducted a search of five electronic databases to identify all relevant articles published between January 2000 and January 2022, and explored the websites of sexual and reproductive health organizations and grey literature databases for unpublished reports. Articles were screened and assessed for eligibility. Included studies were classified and charted according to study location, study aim, study design, type of contraceptive method(s), study population, type of stigma, and intervention approach.

Results

A total of 18 articles were included in the final analysis (11 quantitative, 6 qualitative evaluation methods, and 1 mixed methods). Fourteen of the studies focussed exclusively on abortion stigma, and 2 studies focussed on contraception stigma only. The remaining two, considered both. Among the studies, a majority aimed to address intrapersonal stigma. We found no interventions designed to address stigma at the structural level. In terms of intervention approaches, seven

For peer review only - http://bmjopen.bmj.com/site/about/guidelines.xhtml

were categorized as education/training/skills building, five as counselling/peer support, three as contact, and three as media.

Conclusion

There is a dearth of articles of evaluations of interventions to reduce contraception and abortion stigma. Investment in implementation science is necessary to develop the evidence base and inform the development of effective interventions, and use existing stigma scales to evaluate effectiveness.

For peer review only

For peer review only - http://bmjopen.bmj.com/site/about/guidelines.xhtml

Summary box

What is already known?

- Contraceptive and abortion stigma have a range of health and non-health related consequences.
- Contraceptive and abortion stigma manifest at various levels of society, including the individual, community, provider and institutional level.
- Systematic reviews of interventions to reduce contraception and abortion stigma are non-existant.

What are the new findings?

- Published research about interventions to reduce contraceptive and abortion stigma is limited; this scoping review only located 18 studies.
- Most of these interventions (16) were focussed on addressing abortion stigma.
- Among stigma reduction approaches, the interventions included in this scoping review commonly offered education, training and skills building, and focussed on reducing intrapersonal and interpersonal stigma.

What do the new findings imply?

- There is a need for the development and evaluation of additional interventions to reduce contraceptive and abortion stigma.

For peer review only - http://bmjopen.bmj.com/site/about/guidelines.xhtml
For peer review only

- In particular, there is a significant gap in the availability of interventions to reduce contraceptive and abortion stigma at the structural level and using approaches involving institutions, and laws and policies.
- Future research should assess the effectiveness and scalability of interventions for reducing stigma and discrimination at all levels in the society against women's reproductive health choices.

For peer review only

For peer review only - http://bmjopen.bmj.com/site/about/guidelines.xhtml

Introduction

Health-related stigma has important implications for medical, social, and psychological wellbeing of those affected[1, 2, 3]. In reproductive health, stigmatizing attitudes towards people receiving or providing care is mediated by perceived alignment of practices surrounding human reproduction with societal morals and expectations[4,5]. Contraception and abortion stigma have a range of health and non-health related consequences, such as unintended pregnancy, perceptions of promiscuity, criminalization, secrecy, unsafe abortion and associated complications, and poor quality of abortion care [6, 7]. Contraceptive use stigma emerges where there is censure of the control of fertility, and where contraceptive use conflicts with social norms regarding the life course timing and role of sexual activity, and desired family size [7, 8, 9]. Although rates vary by country[10], abortion is a routine component of sexual and reproductive health. Abortion stigma creates challenges and barriers to access to safe services that can result in deleterious physical, economic and social consequences for women[11, 12].

Abortion stigma has been defined as, "…a negative attribute ascribed to women who seek to terminate a pregnancy that marks them, internally or externally, as inferior to the ideals of womanhood"[13]. Abortion stigma is located and manifests in various aspects of society and affects a wide range of groups[14]. Kumar and colleagues[13] adapted a framework[15] to illustrate the levels of society at which abortion stigma operates and where interventions are needed. At the governmental/structural level abortion stigma is thought to operate in educational, economic, legal, and health policies that reflects social norms discrediting abortion. Internal procedures and

For peer review only - http://bmjopen.bmj.com/site/about/guidelines.xhtml

guidelines that perpetuate abortion stigma characterize the institutional level. At the community level, abortion stigma influences social factors, attitudes and actions that cause individuals involved with abortion to lose status and social networks. Individual-level stigma, or the internalization of negative feelings, such as shame and guilt associated with the experience of abortion, manifests as intrapersonal and interpersonal stigma[13].

Eliminating stigma in sexual and reproductive health care is viewed as critical to avoid negative outcomes and for overall public health[16]. A recent scoping review explored the ways in which abortion stigma influences quality in abortion care[17] and determined that stigma reducing interventions are important for improving quality and decreasing unsafe abortion. A systematic review of multi-level health stigma reduction interventions concluded that research should expand the groups targeted[18]. Although, interventions to reduce contraception and abortion stigma exist[19], the volume, approaches employed, levels targeted, and effectiveness of these remain largely unknown.

We undertook a scoping review to improve understanding of this evidence base. A scoping review approach is appropriate for such indications[20, 21] and is a first step to be followed by assessments of effectiveness in systematic review. The aim of this scoping review was to identify the types, volume and characteristics of available evidence and analyze gaps in the knowledge base for interventions to reduce contraception and abortion stigma.

Methods

For peer review only - http://bmjopen.bmj.com/site/about/guidelines.xhtml

This review was conducted in accordance with Arskey and O'Malley's scoping review methodology and Levac and colleagues' methodological enhancement[22, 23]. We report the methodology and findings according to PRISMA (Preferred Reporting Items for Systematic Reviews and Meta-Analyses) guidelines[24] (Appendix 1).

Search terms

To locate literature for this scoping review, we developed a search strategy with support from experienced librarians in the World Health Organization (WHO) library (Appendix 2). We conducted this search in five electronic databases: MEDLINE, PubMED, EMBASE, Web of Science and PsychINFO to identify articles published between January 2000 and January 2022. We used the Covidence tool (Covidence Systematic Review Software, Veritas Health Innovation, Melbourne, Australia, available at www.covidence.org) to screen, review, organize and extract information from the articles.

Inclusion criteria

We used following eligibility criteria for inclusion in the scoping review: (1) published between January 2000 and January 2022, (2) written in English, (3) reports on an intervention specifically designed to reduce contraceptive and/or abortion stigma in any relevant population as part of the

For peer review only - http://bmjopen.bmj.com/site/about/guidelines.xhtml

primary aim of the study, (4) uses any type of study design, and (5) was conducted in any country context.

Exclusion criteria

We excluded titles and abstracts if they were a review of other research[21] , correction, commentary, editorial, letter, case report, or guideline. Furthermore, we excluded articles that were unavailable through institutional holdings.

Data extraction

Two researchers independently screened the titles and abstracts against the inclusion and exclusion criteria. The full texts deemed relevant by both reviewers were retrieved and reviewed. Two researchers independently assessed the full-text articles to confirm eligibility and reconcile any discrepancies. We additionally conducted a search for grey literature of evaluations of interventions to reduce contraceptive and abortion stigma by exploring the websites of key organizations working in sexual and reproductive health and databases designed to extract unpublished literature, but located no relevant texts during this process.

Data charting process and analysis

For peer review only - http://bmjopen.bmj.com/site/about/guidelines.xhtml

We developed a data-charting form to extract relevant information to answer our scoping review question. One researcher classified and charted all studies according to study location, study aim, study design, type of contraceptive method(s), study population, type of stigma, and intervention approach (Supplementary Table 1). The second researcher reviewed to 20 percent of extractions of included articles to identify any discrepancies. Both researchers met to discuss, re-review articles and resolve any conflicts.

For the analysis of the included literature, we drew upon an existing stigma intervention framework outlined in a 2013 White Paper[19, 25] including the following categories: counselling approaches, information-based education, skills-building education, and contact with effected groups. The interventions included in the four categories aim to address intrapersonal and interpersonal stigma (Table 1). The White Paper included an additional three categorizes for interventions designed to address stigma at the structural level: institutions, laws and policies, and the media[19]. We used this overall framework to organize the literature included in this scoping review. Finally, study quality was not assessed, as this is not a requirement for scoping reviews[21, 22].

Table 1. Categories of analysis

Category	Definition
Type of stigma[13,15]	
Intrapersonal	Internalization of negative feelings, such as shame and guilt, associated with experience with abortion

For peer review only - http://bmjopen.bmj.com/site/about/guidelines.xhtml

Interpersonal	Direct or enacted forms of stigma, such as verbal harassment and/or physical assault based on association with abortion
Community	Social factors and attitudes and actions that cause individuals involved with abortion to lose status in their communities and social networks
Organizational/Institutional	Procedures and guidelines internal to organizations that perpetuate abortion stigma
Governmental/Structural	Educational, economic, legal, health policies and other regulation that reflects social norms that discredit abortion
Intervention approach[19]	
Counselling/peer support	Provide healthcare information, emotional support, and empowerment to people who experience stigma
Education/training/skills building	Provision of information about the stigmatized group and their concerns with the goal of reducing stigma
Contact	In-person interactions that can lead to reductions in prejudice between groups
Protest	An action that publicly calls attention to stigmatizing attitudes and/or behaviours that promote these attitudes
Social marketing	A strategy that uses marketing techniques to achieve behavioural and/or health-related goals
Media	Extended forms of contact that may involve fictional and nonfictional media or visualizations

For peer review only - http://bmjopen.bmj.com/site/about/guidelines.xhtml

Patient and public involvement

As this was a review of exisiting literature, there were no opportunities to engage with the public during its development.

Findings

Our initial search resulted in 16,096 records. After removing 4,192 duplicate articles, two researchers independently screened 11,904 titles and abstracts against the inclusion and exclusion criteria (Figure 1). The full texts of the 196 titles and abstracts were retrieved and reviewed. Of these, 178 were excluded (Figure 1). The remaining 18 texts were included in this review (Figure 1).

Characteristics of included articles

The 18 included studies were conducted in 10 countries: Cambodia (1), Colombia (1), India (1), Indonesia (1), Kenya (1), Mexico[*] (3), South Africa (1), Spain (1), and the United States of America (USA)(8). One study included unspecified countries in the Latin America and Caribbean region and Sub-Saharan Africa. Of the 18 included studies, 11 reported on a quantitative evaluation, 6 reported on a qualitative evaluation, and 1 used mixed methods approaches. Fourteen of the

[*] One study was conducted in Mexico City.

For peer review only

For peer review only - http://bmjopen.bmj.com/site/about/guidelines.xhtml

studies focussed exclusively on abortion stigma, and two studies focussed on contraception stigma. The remaining two considered both. A majority aimed to address intrapersonal stigma. We found no interventions designed to address stigma at the structural level. Table 1 presents the full list of included studies.

Description of stigma reduction interventions

Of the 18 included papers, seven were categorized as education/training/skills building, five were categorized as counselling/peer support, three as contact, and three as media (Table 1). Each intervention is described by category below.

Education/training/skills building

Values clarification is a training intervention for addressing thoughts, feelings and attitudes towards issues that are emotive, such as contraception and abortion. The first article, published In 2015, was an evaluation of the effectiveness of the *Difficult Dialogues* strategies in reducing negative attitudes among midwives in South Africa[26]. The qualitative study suggested that the intervention had some influence on increasing participants' empathy for their patients[26]. The second article evaluated the TEACH (Teaching Everything About Contraceptive Health) program which aimed to dispel myths about contraception and abortion among obstetrican-gynocology residents in a faith-based hospital in the USA. The results of the evaluation suggested that the intervention had limited impact on reducing discriminatory attitudes towards patients seeking abortion services[27]. Finally, a recent evaluation of another values clarification workshop

For peer review only

conducted with residents in Catholic hopsitals in the USA found that, when presented with abortion related-scenarios, residents held less stigmatizing attitudes [28].

Researchers in Spain implemented an intervention for nursing students including a simulation of family planning counselling session. Using a quasi-experimental design, the evaluation determined that two weeks after the intervention, students' attitudes about contraceptive methods improved as measured by relevant validated scales[29].

An evaluation of an intervention conducted in India aimed to empower women to seek services and to normalize abortion and the provision of support by the community and family members of women who seek services. Via a quasi-experimental approach, a scale was used to assess attitudes towards abortion following the intervention. The results suggested that it increased perceived levels of social support for abortion within their families and self-efficacy around family planning and abortion. However, perceptions of support for abortion within communities were lower after the intervention[30].

The Family Planning Balance Counselling Strategy (FP-BCS) is an intervention designed to support the uptake of modern contraceptive methods. Researchers evaluated the effectiveness of the FP-BCS on attitudes, subjective norms and intentions to use modern contraception in Indonesia. An evaluation using a quasi-experimental design determined a significant increase in positive attitudes towards modern contraception among mothers as a result[31].

For peer review only - http://bmjopen.bmj.com/site/about/guidelines.xhtml

An evaluation of a school-based intervention assessed whether Comprehensive Sexuality Education reduced stigma related to abortion and contraceptive use in western Kenya. Among students ages 13-21 at follow-up, stigma scores for both contraception and abortion reduced significantly among boys and girls[32].

Researchers developed a narrative intervention that promoted cognative restructuring and was designed to normalize the abortion experience using a animated 4-minute short-film. People seeking abortion viewed the animation in the waiting room before receiving care. A randomized controlled trial assessed changes to internalized stigma as a result of the intervention. At two-week follow-up there was no significant difference internalized stigma between intervention and control groups[33].

Counselling/peer support

The Story Circles Project intervention was implemented in Mexico and designed to reduce intrapersonal stigma among women who had experienced an abortion. The Story Circles Project provided a safe space for women to share their abortion stories, connect with other women and explore their abortion decision. The intervention also included information sessions to dispel myths and misinformation. The authors found that the intervention significantly reduced women's feelings of isolation helped reduce stigma[34].

Three evaluations of the Provider Share Workshop, an intervention designed for health workers involved in abortion care, were included in this review[35, 36, 37]. The workshop sessions offer

For peer review only

providers an opportunity to discuss and explore their experiences. The 2011 pilot study found that participants reported an “increased interpersonal connection” with the team following the workshop[35]. In 2014, the Provider Share Workshop was conducted among providers in the USA and effectiveness was evaluated using two validated scales. Although the evaluation could not show statistically significant results, the study identified a downward trend in abortion stigma overtime[36]. Finally, the Workshop was adapted and piloted in two countries in Sub-Saharan Africa and Latin America. The evaluation found decreases in overall abortion stigma, but specifically in disclosure management, internalized stigma and judgement[37].

A qualitative assessment of the role of counselling within legal abortion services offered in non-governmental organizations in Mexico City and Colombia in addressing women’s concerns and fears prior to receiving abortion care found that women experienced relief, and less guilt and internalised stigma after counselling[38].

Contact

In 2018, researchers assessed the effectiveness of an intervention designed to reduce interpersonal abortion stigma manifesting as secrecy among women who have experienced abortion and negative reactions from women who learn about the abortion experiences of others. The intervention used the safe spaces of exisiting book clubs to assess whether women would self-disclose their abortion experiences in a positive face-to-face interaction. Book clubs were recruited to read and discuss the book *Choice: True Stories of Birth, Contraception, Infertility, Adoption, Single Parenthood and Abortion*. During the book club intervention, a majority of women

For peer review only

with abortion histories disclosed their experience and the book “warmed” participants feelings about abortion over time[39].

The online platform, Lightbulbs (*Focos*, in Spanish), was designed in Mexico to provide a safe space for women to tell their abortion stories and to increase abortion visibility. In discourse analysis of the text, researchers found that most of the stories were positive and described as a “cathartic” experience[40].

Media

In 2016, researchers examined in the impact of the viewing of the documentary film, *After Tiller*, in reducing third trimester abortion stigma. Viewing the film appeared to challenge myths and misperceptions about abortion. However, the authors found that the medium was limited in its ability to generate new belief systems around abortion[41].

The results of an online study of viewers on an episode of the USA medical television program *Grey's Anatomy* which featured an abortion storyline titled “Papa Don't Preach”. The findings suggested that the program did not increase viewers willingness to support a friend seeking an abortion, whether medically necessary or induced[42].

Discussion

For peer review only - http://bmjopen.bmj.com/site/about/guidelines.xhtml

Despite calls for the development of interventions focussed on reducing reproductive health stigmas, and specifically, contraceptive and abortion stigma, their remains significant gaps in the literature[4, 16, 17, 19]. Most articles identified were reports of evaluations of abortion stigma interventions taking place in the USA. A handful of articles focussed on Latin America, two in Sub-Saharan Africa, one in Asia and one in Europe demonstrating limitations of understanding of available interventions in specific regions and contexts.

The published interventions largely focussed on addressing intrapersonal and interpersonal stigma. The most common intervention approach identified was education, training and skills building. The least common was the contact approach. There is a significant gap in the availability of interventions to reduce contraceptive and abortion stigma at the structural level and using approaches involving institutions, and laws and policies.

There exists substantial theory about intervention approaches that should have an effect on reducing contraception and abortion stigma[1, 15, 16, 19]. Several rigoursly validated scales are available for measuring changes in contraception and abortion stigma. Program developers and evaluators should design and test interventions based on theory and measure their effectiveness using existing validated scales. Future research may synthesize the existing evidence to understand the effectiveness of specific intervention approaches.

The methodology of this scoping review has some limitations. We focused on studies that had an element of evaluation. This meant that we excluded descriptions of activities taking place to

For peer review only

reduce contraception and abortion stigma without efforts to measure effectiveness, acceptability or implementation. This methodological choice may have limited our engagement with interventions currently in use, but not evaluated. It is also possible that evaluators have not published the results of studies of ineffective or inconclusive interventions.

Conclusion

In the design of interventions, stigma continues to be largely conceptualised as an attribute and interventions to mitigate stigma targeted to manifestations at the individual level. Any interventions aligning with this concept may inadvertently (re)generate stigma and minimize the impact of mitgation efforts. Further attention in the development of interventions targeted to the social processes that underpin stigma may have a more profound impact on attitudes and behaviours across populations[43]. Furthermore, efforts to reduce contraceptive and abortion stigma could draw upon a broader stigma framework and benefit from shared concepts, tools, indicators and outcomes[44].

Acknowledgements

The authors would like to acknowledge the input of Kavita Kothari, World Health Organization Librarian, Geneva, Switzerland for her suggestions and support in refining the search strategy.

Disclaimer

The views expressed in this article are those of the authors and do not necessarily represent the views of, and should not be attributed to, the World Health Organization.

For peer review only - http://bmjopen.bmj.com/site/about/guidelines.xhtml

Funding

The UNDP-UNFPA-UNICEF-WHO-World Bank Special Programme of Research, Development and Research Training in Human Reproduction (HRP), a co-sponsored program executed by the World Health Organization (WHO), funded this work.

Competing interests None declared.

Patient consent for publication Not required

Provenance and peer review Not commissioned; externally peer reviewed.

Data availability statement All data relevant to the study are included in the article or uploaded as supplementary information.

Ethics approval An ethics statement is not applicable because this study was based exclusively on published literature.

Contributorship statement AMS and URL conceptualized and conducted the review, including assessing literature and data synthesis. AMS prepared the manuscript with intellectual contributions from URL. Both authors approved the final manuscript.

For peer review only

References

1. Scrambler G, Heijnders M and van Brakel WH on behalf of ICRAAS. (2006) Understanding and tackling health-related stigma. Psychology, Health & Medicine, 11:3, 269-270, DOI: 10.1080/13548500600594908

2. Hatzenbuehler ML, Phelan JC and Link BG. (2013) Stigma as a fundamental cause of population health inequalities. American Journal of Public Health, 103(5): 813-21

3. Ohlsson-Nevo E and Karlsson J. (2018) Impact of health-related stigma on psychosocial functioning in the general population: Construct validity and Swedish reference data for the Stigma-related Social Problems scale (SSP). Research in Nursing and Health, 42: 72-82.

4. Cook RJ and Dicken BM (2014) Reducing stigma in reproductive health. International Journal of Gynecology and Obstetrics, 125: 89-92.

5. Makenzius M, McKinney G, Oguttu M. et al. Stigma related to contraceptive use and abortion in Kenya: scale development and validation. Reprod Health 16, 136 (2019). https://doi.org/10.1186/s12978-019-0799-1

6. Ostrach B (2012) The tangled web of reproductive morbidity risk: Abortion stigma, safety and legality. Frontiers in Women's Health 1 (2)

7. Rehnström Loi U, Otieno B, Oguttu M, Gemzell-Danielsson K, Klingberg-Allvin M, Faxelid E and Makenzius M (2019) Abortion and contraceptive use stigma: a cross-sectional study of attitudes and beliefs in secondary school students in western Kenya. Sexual and Reproductive Health Matters, 27:3, 20-31, DOI: 10.1080/26410397.2019.1652028

8. Blodgett M, Weidert K, Nieto-Andrade B, and Prata N. (2018) Do perceived contraception attitudes influence abortion stigma? Evidence from Luanda, Angola. Social Science and Medicine - Population Health; 5: 38-47.

For peer review only

9. Farmer DB, Berman L, Ryan G, Habumugisha L, Basinga P, Nutt C, Kamali F, Ngizwenayo E, St. Fluer J, Niyigena P, Ngabo F, Farmer PE and Rich ML. (2015) Motivations and contraints to family planning: A qualitative study in Rwanda's Southern Kayonza District. Global Health: Science and Practice, 3(2):242-254; https://doi.org/10.9745/GHSP-D-14-00198

10. Bearak JM, Popinchalk A, Beavin C, Ganatra B, Moller AB, Tunçalp Ö, and Alkema L. (2022) Country-specific estimates of unintended pregnancy and abortion incidence: a global comparative analysis of levels in 2015–2019. BMJ Global Health;7:e007151. doi:10.1136/
bmjgh-2021-007151

11. Hanschmidt F, Linde K, Hilbert A, Riedel-Heller SG and Kersting A (2016) Abortion stigma: A systematic review. Perspectives on Sexual and Reproductive Health, 48(4): doi: 10.1363/48e8516

12. Moore B, Poss C, Coast E, Lattof SR, van der Meulen Rodgers Y (2021) The economics of abortion and its links with stigma: A secondary analysis from a scoping review on the economics of abortion. PLoS ONE 16(2): e0246238. https://doi.org/10.1371/journal.pone.0246238

13. Kumar A, Hesseni L and Mitchell EMH (2009) Conceptualising abortion stigma. Culture, Health and Sexuality, Aug;11(6):625-39. doi: 10.1080/13691050902842741.

14. Norris A, Bessett D, Steinberg JR, Kavanaugh ML, De Zordo S, and Becker D. (2011) Abortion stigma: A reconceptualization of constituents, causes, and consequences. Women's Health Issues, doi:10.1016/j.whi.2011.02.010

15. Heijinders M and van Der Meij (2006) The fight against stigma: an overview of stigma-reduction strategies and interventions. Psychol Health Med, Aug;11(3):353-63. doi: 10.1080/13548500600595327

For peer review only

16. Hussein J and Ferguson L (2019) Eliminating stigma and discrimination in sexual and reproductive health care: a public health imperative, Sexual and Reproductive Health Matters, 27:3, 1-5, DOI: 10.1080/26410397.2019.1697103

17. Sorhaindo A and Lavelanet AF. Why does abortion stigma matter? A scoping review and conceptual framework describing the role of stigma in shaping abortion quality of care, under development

18. Rao D, Elshafei A, Nguyen M, Hatzenbuehler ML, Frey S and Go VF. (2019) A systematic review of multi-level stigma interventions: state of the science and future directions. 17:41 https://doi.org/10.1186/s12916-018-1244-y

19. Cockrill K, Herold S, Blanchard K, Grossman D, Upadhyay U, Baum S. (2013a). Addressing Abortion Stigma Through Service Delivery: A White Paper. Retrieved from Ibis Reproductive Health: https://www.ibisreproductivehealth.org/publications/addressing-abortion-stigma-through-service-delivery-white-paper.

20. Armstrong R, Hall BJ Doyle J and Waters E. (2011) “Scoping the scope” of a cochrane review. Journal of Public Health, 33(1): 147-150.

21. Munn Z, Peters MDJ, Stern C, Tufanaru, McArthur A and Aromataris E. (2018) Systematic review or scoping review? Guidance for authors when choosing between a systematic or scoping review approach. BMC Medical Research Methodology, 18:143, https://doi.org/10.1186/s12874-018-0611-x

22. Arksey, H and O'Malley, L (2005) Scoping studies: towards a methodological framework. International Journal of Social Research Methodology, 19-32.

23. Levac D, Colquhoun H, O'Brien KK. (2010) Scoping studies: advancing the methodology. Implement Sci, 5:69.

For peer review only

24. Moher D, Shamseer L, Clarke M, Ghersi D, Liberati A, Petticrew M et al. (2015) Preferred reporting items for systematic review and meta-analysis protocols (PRISMA-P) 2015 statement. Syst Rev, 4: 1 https://doi.org/10.1186/2046-4053-4-1

25. Brown, L., Macintyre, K., & Trujillo, L. (2003). Interventions to reduce HIV/AIDS stigma: What have we learned? AIDS Education and Prevention, 15(1), 49-69.

26. Mpeli MR and Botma Y (2015) Abortion-related services: Values clarification through 'Diffcult Dialogues' strategies. Education, Citizenship and Social Justice, 10(3): 278-288

27. Guiahi M, Cortland C, Graham MJ, Heraty S, Lukens M, Trester M, Summers S, Kenton K (2011) Addressing OB/GYN family planning educational objectives at a faith-based institution using the TEACH program. Contraception, 83: 367-372.

28. Guiahi M, Wilson C, Claymore E, Simonson K, Steinauer J. (2021) Influence of a values clarification workshop on residents training at Catholic Hospital programs. Contraception: X 3 100054

29. Sanz-Martos S, López-Medina IM, Álvarez-Garcia C, Álvarez-Nieto C. (2021) Educational program on sexuality and contraceptive methods in nursing degree students. Nurse Education Today, 107: 105114

30. Banerjee SK, Andersen KL, Warvadekar J and Pearson E. (2013) Intervention to improve knowledge and perceptions abortion abortion in Bihar and Jharkhand, India. International Perspectives in Sexual and Reproductive Health, 39(3): 142-151, doi:10.1363/3914213

31. Palinggi RS, Moedjiono AI, Suarayasa K, Masni, Seweg A, Amqam H, Nur R amd Syam A (2021) The effect of balanced counseling strategy family planning against attitude, subjective norm, and intentions on the use of modern contraception behavior in the Singgani Public Health Center work area of Palu city. Gac Sanit

For peer review only

32. Rehnström Loi U. (2020) Abortion, contraception and associated social stigma – consequences and solutions in a low-resource setting in Western Kenya. PhD Thesis. Karolinska Institutet, Stockholm, Sweden.

33. Sackeim MG, Lee SY, Newton S, Lauderdale D, Gilliam M (2022). Effect of a narrative intervention on individual-level abortion stigma: a randomised controlled trial. BMJ Sexual and Reproductive Health; 48:47–53.

34. Belfrage M, Ortíz Ramírez O and Sorhaindo A. (2019) Story Circles and abortion stigma in Mexico: a mixed-methods evaluation of a new intervention for reducing individual level abortion stigma, Culture, Health & Sexuality, DOI: 10.1080/13691058.2019.1577493

35. Harris LH, Debbink M, Martin L, and Hassinger J (2011) Dynamics of stigma in abortion work: Findings from a pilot of the Providers Share Workshop. Social Science and Medicine, 73: 1062-1070.

36. Martin LA, Debbink M, Hassinger J, Youatt E and Harris LH. (2014) Abortion providers, stigma and professional quality of life. Contraception, 90: 581-587.

37. Mosley EA, Martin L, Seewald M, Hassinger J, Blanchard K, Baum SE, Santana D, Echeverri L, Garrett J, Njunguru J and Harris L (2020) International Perspectives on Sexual and Reproductive Health, 46: 35-50.

38. Keefe-Oates B, Makleff S, Sa E, Forero LJ, Mendoza D, Olaya MA. (2019) Experiences with abortion counselling in Mexico City and Colombia: addressing women's fears and concerns. Culture, Health and Sexuality, 4.

39. Cockrill K and Biggs A. (2018) Can stories reduce abortion stigma? Findings from a longitudinal cohort study. Culture, Health and Sexuality, 3.

40. Belfrage M, Didier E and Vázquez-Quesada L (2021): Voicing Abortion Experiences to Reduce Stigma: Lessons from an Online Storytelling Platform in Mexico, Women's Reproductive Health, DOI: 10.1080/23293691.2021.2016145

For peer review only

41. Sisson G and Kimport K (2016) After After Tiller: the impact of a documentary film on understandings of third-trimester abortion. Culture, Health and Sexuality: 6.

42. Sisson G, Walter N, Harold S and Brooks JJ. (2021) Prime-time abortion on Grey's Anatomy: What do US viewers learn from fictional portrayals of abortion on television?

43. Millar E. (2020) Abortion stigma is a social process. Women's Studies International Forum, 78: 102328

44. Stangl AL, Earnshaw VA, Logie CH, van Brakel W, Simbayi LC, Barré I and Dovidio JF. (2019) The Health Stigma and Discrimination Framework: a global, crosscutting framework to inform research, intervention development, and policy on health-related stigmas. BMC Medicine, 17:31

Figure legend

Figure 1. Scoping review PRISMA flowchart

For peer review only

For peer review only

For peer review only - http://bmjopen.bmj.com/site/about/guidelines.xhtml

For peer review only

For peer review only - http://bmjopen.bmj.com/site/about/guidelines.xhtml

Figure 1. Scoping review PRISMA flowchart

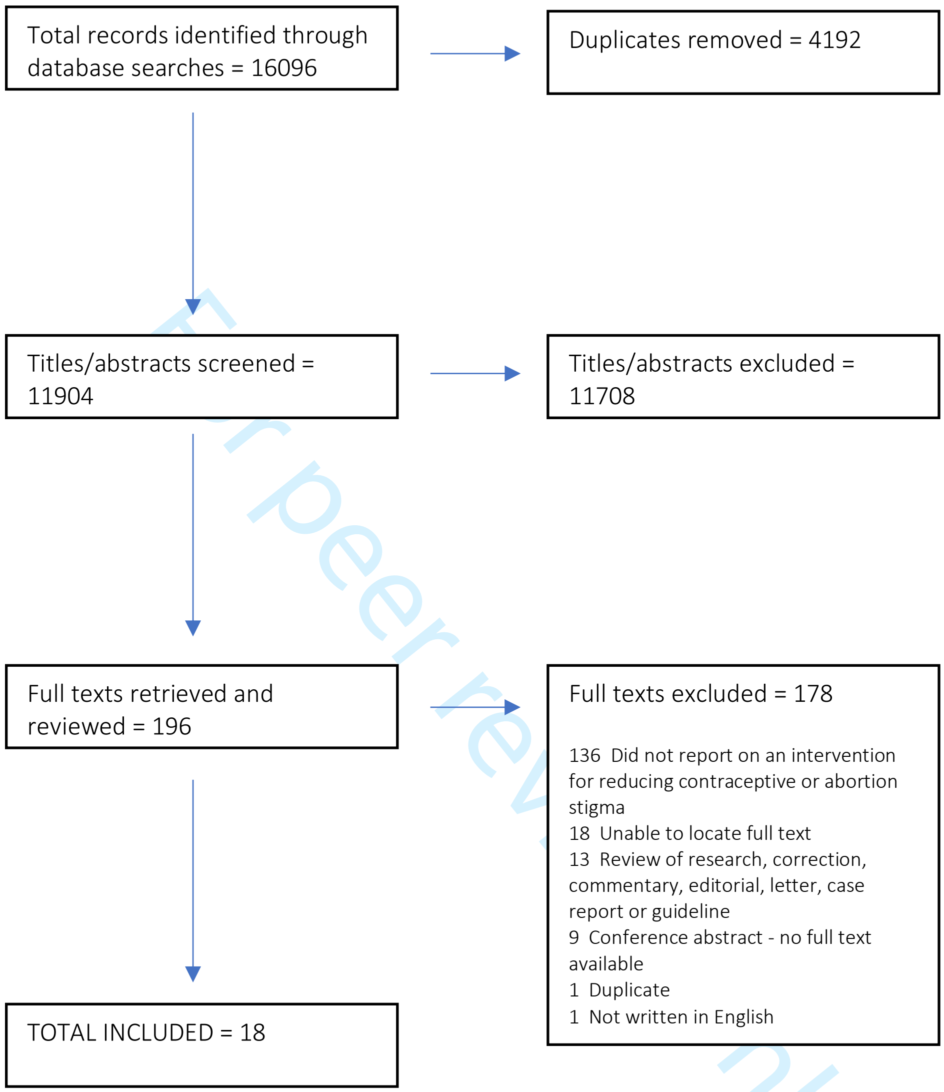

For peer review only

For peer review only - http://bmjopen.bmj.com/site/about/guidelines.xhtml

Supplemental material

Supplementary Table 1. Characteristics of included studies (n=18)

Author	Year of publication	Country	Scope	Evaluation design	Focus	Type of stigma	Intervention approach
Banerjee	2013	India	To evaluate the effectiveness of a behavior change communication intervention to improve women's knowiedge about India's abortion law and their perceptions about abortion	Quasi-experimental	Abortion	Community	Education/training/skills building
Belfarge	2020	Mexico	The evaluation measured whether participation in the intervention reduced women’s experience of individual level stigma one month after having participated and explored women’s expressed experiences of the intervention and their perceptions of changes in stigmatising feelings after the intervention.	Mixed methods	Abortion	Intrapersonal	Counselling/peer support
Belfarge	2021	Mexico	To understand more about who tells their stories online, how they represent their abortion experience, and what lessons this leaves us as feminist activists seeking to advance sexual and reproductive rights through online storytelling	Qualitative	Abortion	Intrapersonal	Contact
Cockrill	2018	USA	To measure changes in women's attitudes toward abortion and their willingness to disclose their own abortion experiences with one another	Quasi-experimental	Abortion	Interpersonal	Contact

For peer review only

			following a book group discussion in which participants discussed a previously chosen book.				
Guiahi	2011	USA	To evaluate if a values clarification workshop conducted at Catholic hospital training programs influenced obstetrics and gynecology residents' abortion attitudes.	Quasi-experimental	Abortion	Interpersonal	Education/training/skills building
Guiahi	2021	USA	To understand whether a values clarification workshop influences abortion attitudes of obgyn residents at U.S. training programs that are based in Catholic hospitals and lack abortion training.	Quasi-experimental	Abortion	Interpersonal	Education/training/skills building
Harris	2011	USA	To present findings from a qualitative pilot study of the Providers Share Workshop, a six-session workshop in which abortion providers (broadly defined to include anyone with direct daily involvement in abortion care), meet to explore their experiences, guided by an experienced facilitator	Qualitative	Abortion	Intrapersonal	Counselling/peer support
Keefe-Oates	2020	Colombia and Mexico City	To develop a greater understanding of their clients' concerns prior to abortion care,and the role that counselling can play in addressing these concerns.	Qualitative	Abortion	Intrapersonal	Counselling/peer support
Martin	2014	USA	The Providers Share Workshop (PSW) provides abortion providers safe space to discuss their work experiences. Our	Quasi-experimental	Abortion	Intrapersonal	Counselling/peer support

For peer review only

			objectives were to assess changes in abortion stigma over time and explore how stigma is related to aspects of professional quality of life, including compassion satisfaction, burnout and compassion fatigue for providers participating in the workshops.				
Mosley	2020	Sub-Saharan Africa and Latin American and the Caribbean*	To adapt the Providers Share Workshop content, structure and evaluation tools for this pilot study in Sub-Saharan Africa and Latin America.	Cohort	Abortion	Intrapersonal	Counselling/peer support
Mpeli	2015	South Africa	To report on the value clarification process and the views of preregistration midwifery students regarding abortion-related services before and after implementing the value clarification process.	Qualitative	Abortion	Intrapersonal	Education/training/skills building
Palinggi	2021	Indonesia	To analyze the influence of the Balanced Counseling Strategy (FP-BCS) on attitudes, subjective norms, and intentions on modern contraception in the working area of Singgani Health Center, Palu, Indonesia	Quasi-experimental	Contraception	Community	Education/training/skills building
Rehnström Loi	2020	Kenya	To measure attitudes towards abortion and contraceptive use, and to evaluate a stigma-reduction intervention among secondary school students.	Quasi-experimental	Both	Interpersonal	Education/training/skills building

For peer review only

Sackeim	2022	USA	To evaluate the effect of a narrative intervention on individual-level abortion stigma in patients undergoing abortion.	Randomized Controlled Trial	Abortion	Intrapersonal	Contact
San-Martos	2021	Spain	This study aimed to evaluate the effect of a peer-educational intervention to improve knowledge level and attitudes regarding contraceptive methods in university students.	Quasi-experimental	Contraception	Community	Education/training/skills building
Sisson	2016	USA	To determine whether a documentary film can impact stigmatizing attaitudes against 3rd trimester abortion.	Qualitative	Abortion	Community	Media
Sisson	2021	USA	To examine the impact of an abortion storyline from the TV show Grey's Anatomy on USA-based viewers.	Quasi-experimental	Abortion	Community	Media
Smith	2021	Cambodia	The aim of this study was to describe the development of an intervention to support the reproductive health of garment factory workers in Cambodia	Qualitative	Both	Community	Media

*Only regions were report

For peer review only

Appendix 1. Preferred Reporting Items for Systematic reviews and Meta-Analyses extension for Scoping Reviews (PRISMA-ScR) Checklist

SECTION	ITEM	PRISMA-ScR CHECKLIST ITEM	REPORTED ON PAGE #
TITLE			
Title	1	Identify the report as a scoping review.	1
ABSTRACT			
Structured summary	2	Provide a structured summary that includes (as applicable): background, objectives, eligibility criteria, sources of evidence, charting methods, results, and conclusions that relate to the review questions and objectives.	2
INTRODUCTION			
Rationale	3	Describe the rationale for the review in the context of what is already known. Explain why the review questions/objectives lend themselves to a scoping review approach.	4
Objectives	4	Provide an explicit statement of the questions and objectives being addressed with reference to their key elements (e.g., population or participants, concepts, and context) or other relevant key elements used to conceptualize the review questions and/or objectives.	4
METHODS			
Protocol and registration	5	Indicate whether a review protocol exists; state if and where it can be accessed (e.g., a Web address); and if available, provide registration information, including the registration number.	N/A
Eligibility criteria	6	Specify characteristics of the sources of evidence used as eligibility criteria (e.g., years considered, language, and publication status), and provide a rationale.	5
Information sources*	7	Describe all information sources in the search (e.g., databases with dates of coverage and contact with authors to identify additional sources), as well as the date the most recent search was executed.	5
Search	8	Present the full electronic search strategy for at least 1 database, including any limits used, such that it could be repeated.	26
Selection of sources of evidence†	9	State the process for selecting sources of evidence (i.e., screening and eligibility) included in the scoping review.	5
Data charting process‡	10	Describe the methods of charting data from the included sources of evidence (e.g., calibrated forms or forms that have been tested by the team before their use, and whether data charting was done independently or in	5-6

For peer review only

SECTION	ITEM	PRISMA-ScR CHECKLIST ITEM	REPORTED ON PAGE #
		duplicate) and any processes for obtaining and confirming data from investigators.	
Data items	11	List and define all variables for which data were sought and any assumptions and simplifications made.	22
Critical appraisal of individual sources of evidence§	12	If done, provide a rationale for conducting a critical appraisal of included sources of evidence; describe the methods used and how this information was used in any data synthesis (if appropriate).	N/A
Synthesis of results	13	Describe the methods of handling and summarizing the data that were charted.	6
RESULTS			
Selection of sources of evidence	14	Give numbers of sources of evidence screened, assessed for eligibility, and included in the review, with reasons for exclusions at each stage, ideally using a flow diagram.	15
Characteristics of sources of evidence	15	For each source of evidence, present characteristics for which data were charted and provide the citations.	16-21
Critical appraisal within sources of evidence	16	If done, present data on critical appraisal of included sources of evidence (see item 12).	N/A
Results of individual sources of evidence	17	For each included source of evidence, present the relevant data that were charted that relate to the review questions and objectives.	Click here to enter text.
Synthesis of results	18	Summarize and/or present the charting results as they relate to the review questions and objectives.	7-9
DISCUSSION			
Summary of evidence	19	Summarize the main results (including an overview of concepts, themes, and types of evidence available), link to the review questions and objectives, and consider the relevance to key groups.	9-10
Limitations	20	Discuss the limitations of the scoping review process.	10
Conclusions	21	Provide a general interpretation of the results with respect to the review questions and objectives, as well as potential implications and/or next steps.	9-10
FUNDING			
Funding	22	Describe sources of funding for the included sources of evidence, as well as sources of funding for the scoping review. Describe the role of the funders of the scoping review.	10

JBI = Joanna Briggs Institute; PRISMA-ScR = Preferred Reporting Items for Systematic reviews and Meta-Analyses extension for Scoping Reviews.
* Where *sources of evidence* (see second footnote) are compiled from, such as bibliographic databases, social media platforms, and Web sites.
† A more inclusive/heterogeneous term used to account for the different types of evidence or data sources (e.g., quantitative and/or qualitative research, expert opinion, and policy

documents) that may be eligible in a scoping review as opposed to only studies. This is not to be confused with *information sources* (see first footnote).
‡ The frameworks by Arksey and O'Malley (6) and Levac and colleagues (7) and the JBI guidance (4, 5) refer to the process of data extraction in a scoping review as data charting.
§ The process of systematically examining research evidence to assess its validity, results, and relevance before using it to inform a decision. This term is used for items 12 and 19 instead of "risk of bias" (which is more applicable to systematic reviews of interventions) to include and acknowledge the various sources of evidence that may be used in a scoping review (e.g., quantitative and/or qualitative research, expert opinion, and policy document).

From: Tricco AC, Lillie E, Zarin W, O'Brien KK, Colquhoun H, Levac D, et al. PRISMA Extension for Scoping Reviews (PRISMAScR): Checklist and Explanation. Ann Intern Med. 2018;169:467–473. doi: 10.7326/M18-0850.

For peer review only

Appendix 2. Example search strategy

	#	Searches
Stigma		Social stigma* OR shame* OR guilt* OR discriminat* OR Prejudice* OR judgement OR stereotype*OR blame OR isolate* OR moral* OR harass* OR insult* OR labeling OR social-accept* OR social-approv* OR social-percep*OR treatment-barrier*
Abortion or contraception		abortion, induced OR Abortion, Criminal OR Abortion, Septic OR Abortion Applicants OR abortion, missed OR abortion, legal OR abortion, spontaneous OR abortion* OR pregnancy-terminat* OR post-abortion*OR Postconception Fertility Control OR Embryotomies OR Embryotomy OR Dilatation and curettage OR Vacuum Curettage OR Surgical abortion OR Manual vacuum aspiration OR (vacuum AND aspiration) OR Curettage OR Surgical termination of pregnancy OR Dilatation and evacuation OR Dilation and evacuation OR Suction aspiration OR Aspiration abortion OR Suction curettage OR Vacuum curettage OR Contraception OR Contraception Behavior OR Condom OR Condoms OR Diaphragm OR Diaphragms OR Cervical Cap OR Vaginal-sponge OR Cervical-Caps OR Vaginal-sponges OR Birth-Control OR Contraceptive-Methods OR Contraceptive-Method OR Female Contraception OR Female Contraceptions OR Male Contraception OR Male Contraceptions OR Inhibition-of-Fertilization OR Fertilization-Inhibition OR Fertility-Control OR Birth-Control OR Vaginal-Barrier OR Hormonal-Contracept* OR anticonception OR antifertility OR conception-control OR family planning OR birth control OR birth-prevention OR pregnancy-prevent*
Program evaluation-related		intervention OR program OR activity OR action

For peer review only

Research Checklist

Appendix 1. Preferred Reporting Items for Systematic reviews and Meta-Analyses extension for Scoping Reviews (PRISMA-ScR) Checklist

SECTION	ITEM	PRISMA-ScR CHECKLIST ITEM	REPORTED ON PAGE #
**TITLE**			
Title	1	Identify the report as a scoping review.	1
**ABSTRACT**			
Structured summary	2	Provide a structured summary that includes (as applicable): background, objectives, eligibility criteria, sources of evidence, charting methods, results, and conclusions that relate to the review questions and objectives.	2
**INTRODUCTION**			
Rationale	3	Describe the rationale for the review in the context of what is already known. Explain why the review questions/objectives lend themselves to a scoping review approach.	4
Objectives	4	Provide an explicit statement of the questions and objectives being addressed with reference to their key elements (e.g., population or participants, concepts, and context) or other relevant key elements used to conceptualize the review questions and/or objectives.	4
**METHODS**			
Protocol and registration	5	Indicate whether a review protocol exists; state if and where it can be accessed (e.g., a Web address); and if available, provide registration information, including the registration number.	N/A

For peer review only

SECTION	ITEM	PRISMA-ScR CHECKLIST ITEM	REPORTED ON PAGE #
Eligibility criteria	6	Specify characteristics of the sources of evidence used as eligibility criteria (e.g., years considered, language, and publication status), and provide a rationale.	5
Information sources*	7	Describe all information sources in the search (e.g., databases with dates of coverage and contact with authors to identify additional sources), as well as the date the most recent search was executed.	5
Search	8	Present the full electronic search strategy for at least 1 database, including any limits used, such that it could be repeated.	26
Selection of sources of evidence†	9	State the process for selecting sources of evidence (i.e., screening and eligibility) included in the scoping review.	5
Data charting process‡	10	Describe the methods of charting data from the included sources of evidence (e.g., calibrated forms or forms that have been tested by the team before their use, and whether data charting was done independently or in duplicate) and any processes for obtaining and confirming data from investigators.	5-6
Data items	11	List and define all variables for which data were sought and any assumptions and simplifications made.	22
Critical appraisal of individual sources of evidence§	12	If done, provide a rationale for conducting a critical appraisal of included sources of evidence; describe the methods used and how this information was used in any data synthesis (if appropriate).	N/A
Synthesis of results	13	Describe the methods of handling and summarizing the data that were charted.	6

For peer review only

SECTION	ITEM	PRISMA-ScR CHECKLIST ITEM	REPORTED ON PAGE #
**RESULTS**			
Selection of sources of evidence	14	Give numbers of sources of evidence screened, assessed for eligibility, and included in the review, with reasons for exclusions at each stage, ideally using a flow diagram.	15
Characteristics of sources of evidence	15	For each source of evidence, present characteristics for which data were charted and provide the citations.	16-21
Critical appraisal within sources of evidence	16	If done, present data on critical appraisal of included sources of evidence (see item 12).	N/A
Results of individual sources of evidence	17	For each included source of evidence, present the relevant data that were charted that relate to the review questions and objectives.	Click here to enter text.
Synthesis of results	18	Summarize and/or present the charting results as they relate to the review questions and objectives.	7-9
**DISCUSSION**			
Summary of evidence	19	Summarize the main results (including an overview of concepts, themes, and types of evidence available), link to the review questions and objectives, and consider the relevance to key groups.	9-10
Limitations	20	Discuss the limitations of the scoping review process.	10
Conclusions	21	Provide a general interpretation of the results with respect to the review questions and objectives, as well as potential implications and/or next steps.	9-10
**FUNDING**			

For peer review only

SECTION	ITEM	PRISMA-ScR CHECKLIST ITEM	REPORTED ON PAGE #
Funding	22	Describe sources of funding for the included sources of evidence, as well as sources of funding for the scoping review. Describe the role of the funders of the scoping review.	10

JBI = Joanna Briggs Institute; PRISMA-ScR = Preferred Reporting Items for Systematic reviews and Meta-Analyses extension for Scoping Reviews.
* Where *sources of evidence* (see second footnote) are compiled from, such as bibliographic databases, social media platforms, and Web sites.
† A more inclusive/heterogeneous term used to account for the different types of evidence or data sources (e.g., quantitative and/or qualitative research, expert opinion, and policy documents) that may be eligible in a scoping review as opposed to only studies. This is not to be confused with *information sources* (see first footnote).
‡ The frameworks by Arksey and O'Malley (6) and Levac and colleagues (7) and the JBI guidance (4, 5) refer to the process of data extraction in a scoping review as data charting.
§ The process of systematically examining research evidence to assess its validity, results, and relevance before using it to inform a decision. This term is used for items 12 and 19 instead of "risk of bias" (which is more applicable to systematic reviews of interventions) to include and acknowledge the various sources of evidence that may be used in a scoping review (e.g., quantitative and/or qualitative research, expert opinion, and policy document).

From: Tricco AC, Lillie E, Zarin W, O'Brien KK, Colquhoun H, Levac D, et al. PRISMA Extension for Scoping Reviews (PRISMAScR): Checklist and Explanation. Ann Intern Med. 2018;169:467–473. doi: 10.7326/M18-0850.

For peer review only

For peer review only

BMJ Open

Interventions to reduce stigma related to contraception and abortion: a scoping review

Journal:	*BMJ Open*
Manuscript ID	bmjopen-2022-063870.R1
Article Type:	Original research
Date Submitted by the Author:	14-Sep-2022
Complete List of Authors:	Sorhaindo, Annik; World Health Organization, Sexual and Reproductive Health and Research<br>Rehnstrom Loi, Ulrika; World Health Organization, Sexual and Reproductive Health and Research
**Primary Subject Heading**:	Public health
Secondary Subject Heading:	Reproductive medicine
Keywords:	PUBLIC HEALTH, REPRODUCTIVE MEDICINE, MEDICAL ETHICS

SCHOLARONE™
Manuscripts

I, the Submitting Author has the right to grant and does grant on behalf of all authors of the Work (as defined in the below author licence), an exclusive licence and/or a non-exclusive licence for contributions from authors who are: i) UK Crown employees; ii) where BMJ has agreed a CC-BY licence shall apply, and/or iii) in accordance with the terms applicable for US Federal Government officers or employees acting as part of their official duties; on a worldwide, perpetual, irrevocable, royalty-free basis to BMJ Publishing Group Ltd ("BMJ") its licensees and where the relevant Journal is co-owned by BMJ to the co-owners of the Journal, to publish the Work in this journal and any other BMJ products and to exploit all rights, as set out in our licence.

The Submitting Author accepts and understands that any supply made under these terms is made by BMJ to the Submitting Author unless you are acting as an employee on behalf of your employer or a postgraduate student of an affiliated institution which is paying any applicable article publishing charge ("APC") for Open Access articles. Where the Submitting Author wishes to make the Work available on an Open Access basis (and intends to pay the relevant APC), the terms of reuse of such Open Access shall be governed by a Creative Commons licence – details of these licences and which Creative Commons licence will apply to this Work are set out in our licence referred to above.

Other than as permitted in any relevant BMJ Author's Self Archiving Policies, I confirm this Work has not been accepted for publication elsewhere, is not being considered for publication elsewhere and does not duplicate material already published. I confirm all authors consent to publication of this Work and authorise the granting of this licence.

For peer review only

Title

Interventions to reduce stigma related to contraception and abortion: a scoping review

Authors

Annik Mahalia Sorhaindo, MSc PhD[1], Ulrika Rehnström Loi, MSc PhD[1*]

*Corresponding author: Ulrika Rehnström Loi, 20 Avenue Appia, 1211 Geneva, Switzerland, e-mail: rehnstromu@who.int

1. UNDP-UNFPA-UNICEF-WHO-World Bank Special Programme of Research, Development and Research Training in Human Reproduction (HRP), Department of Sexual and Reproductive Health and Research, World Health Organization, 20 Avenue Appia, 1211 Geneva, Switzerland

Key words (9): abortion stigma, contraceptive use stigma, values clarification, intervention, evaluation

For peer review only

For peer review only - http://bmjopen.bmj.com/site/about/guidelines.xhtml

Word count: 4659/5000 (including summary box, references, tables and figures)

Tables: 2 Figures: 1

For peer review only

For peer review only - http://bmjopen.bmj.com/site/about/guidelines.xhtml

Abstract

Objectives:

We conducted a scoping review to identify the types, volume and characteristics of available evidence and analyze gaps in the knowledge base for evaluated interventions to reduce contraception and abortion stigma.

Methods

We conducted a search of five electronic databases MEDLINE, PubMED, EMBASE, Web of Science and PsychINFO to identify all relevant articles published between January 2000 and January 2022, and explored the websites of sexual and reproductive health organizations and grey literature databases for unpublished and non-commercial reports. Articles were assessed for eligibility. Included studies were charted according to study location, study aim, study design, type of contraceptive method(s), study population, type of stigma, and intervention approach.

Results

Some 18 articles were included in the final analysis (11 quantitative, 6 qualitative evaluation methods, and 1 mixed methods). Fourteen of the studies focussed exclusively on abortion stigma, and 2 studies focussed on contraception stigma only. The remaining two, considered both. Among the studies, a majority aimed to address intrapersonal stigma. We found no interventions designed to address stigma at the structural level. In terms of intervention approaches, seven

For peer review only - http://bmjopen.bmj.com/site/about/guidelines.xhtml

were categorized as education/training/skills building, five as counselling/peer support, three as contact, and three as media.

Conclusion

There is a dearth of articles of evaluations of interventions to reduce contraception and abortion stigma. Investment in implementation science is necessary to develop the evidence base and inform the development of effective interventions, and use existing stigma scales to evaluate effectiveness. This scoping review can serve as a precursor to systematic reviews assessing the effectiveness of approaches.

For peer review only - http://bmjopen.bmj.com/site/about/guidelines.xhtml

Strengths and limitations of this study

- The systematic inclusion of contraceptive stigma in the search exposed a gap in the evidence of interventions designed to address this reproduction health stigma.
  The search strategy developed was designed to capture a broad swath of the literature for review.
- The interventions were organized according to an existing framework for abortion stigma interventions
- We limited our search to studies that were published in English which may have excluded key articles written in other languages.
- We focused on studies that had an element of evaluation, thereby excluding descriptions of activities taking place to reduce contraception and abortion stigma without efforts to measure effectiveness, acceptability or implementation.

For peer review only - http://bmjopen.bmj.com/site/about/guidelines.xhtml

Introduction

Health-related stigma has important implications for medical, social, and psychological wellbeing of those affected [1, 2, 3]. In reproductive health, stigmatizing attitudes towards people receiving or providing care is mediated by perceived alignment of practices surrounding human reproduction with societal morals and expectations [4, 5]. Contraception and abortion stigma have a range of health and non-health related consequences, such as unintended pregnancy, perceptions of promiscuity, criminalization, secrecy, unsafe abortion and associated complications, and poor quality of abortion care [6, 7]. Contraceptive use stigma emerges where there is censure of the control of fertility, and where contraceptive use conflicts with social norms regarding the life course timing and role of sexual activity, and desired family size [7, 8, 9]. Although rates vary by country [10], abortion is a routine component of sexual and reproductive health. Abortion stigma creates challenges and barriers to access to safe services that can result in deleterious physical, economic and social consequences for women [11, 12]. The two phenomenon are rooted in the negation of the right of inidviudals to decide whether and when to have children, make decisions about and have control of their body and sexuality, and manifest similarly in social norms and practices that hinder access to family planning and lead to unintended pregnancy [7].

Abortion stigma has been defined as, "…a negative attribute ascribed to women who seek to terminate a pregnancy that marks them, internally or externally, as inferior to the ideals of womanhood"[13]. Abortion stigma is located and manifests in various aspects of society and

For peer review only - http://bmjopen.bmj.com/site/about/guidelines.xhtml

affects a wide range of groups [14]. Kumar and colleagues [13] adapted a framework [15] to illustrate the levels of society at which abortion stigma operates and where interventions are needed. At the governmental/structural level abortion stigma is thought to operate in educational, economic, legal, and health policies that reflects social norms discrediting abortion. Internal procedures and guidelines that perpetuate abortion stigma characterize the institutional level. At the community level, abortion stigma influences social factors, attitudes and actions that cause individuals involved with abortion to lose status and social networks. Individual-level stigma, or the internalization of negative feelings, such as shame and guilt associated with the experience of abortion, manifests as intrapersonal and interpersonal stigma [13]. Investigation of contraceptive stigma is limited; however, one analysis with adolscents in Kenya determined that negative sterotypes and discrimination results in delays in access to health care and missed opportunities for contraceptive counselling [7, 16].

Eliminating stigma in sexual and reproductive health care is viewed as critical to avoid negative outcomes and for overall public health [17]. A recent scoping review explored the ways in which abortion stigma influences quality in abortion care [18] and determined that stigma reducing interventions are important for improving quality and decreasing unsafe abortion. A systematic review of multi-level health stigma reduction interventions concluded that research should expand the groups targeted [19]. Although, interventions to reduce contraception and abortion stigma exist [20], the volume, approaches employed, levels targeted, and effectiveness of these remain largely unknown.

For peer review only - http://bmjopen.bmj.com/site/about/guidelines.xhtml

We undertook a scoping review to identify the key characteristics of this evidence base and analyze gaps. A scoping review approach is appropriate for such indications [21, 22] and is a first step to be followed by assessments of effectiveness in systematic review. The aim of this scoping review was to identify the types, volume and characteristics of available evidence and analyze gaps in the knowledge base for interventions to reduce contraception and abortion stigma.

Methods

This review was conducted in accordance with Arskey and O'Malley's scoping review methodology and Levac and colleagues' methodological enhancement [23, 24]. We report the methodology and findings according to PRISMA (Preferred Reporting Items for Systematic Reviews and Meta-Analyses) guidelines [25] (Appendix 1).

Search terms

To locate literature for this scoping review, we developed a search strategy with support from experienced librarians in the World Health Organization (WHO) library (Appendix 2). We conducted this search in five electronic databases: MEDLINE, PubMED, EMBASE, Web of Science and PsychINFO to identify articles published between January 2000 and January 2022. We used the Covidence tool (Covidence Systematic Review Software, Veritas Health Innovation, Melbourne, Australia, available at www.covidence.org) to screen, review, organize and extract information from the articles.

For peer review only - http://bmjopen.bmj.com/site/about/guidelines.xhtml

Inclusion criteria

We used following eligibility criteria for inclusion in the scoping review: (1) published between January 2000 and January 2022, (2) written in English, (3) reports on the evaluation of an intervention specifically designed to reduce contraceptive and/or abortion stigma in any relevant population as part of the primary aim of the study, (4) uses any type of study design, and (5) was conducted in any country context.

Exclusion criteria

We excluded titles and abstracts if they were a review of other research [22] , correction, commentary, editorial, letter, case report, or guideline. Furthermore, we excluded articles that were unavailable through institutional holdings.

Data extraction

Two researchers independently screened the titles and abstracts against the inclusion and exclusion criteria. The full texts deemed relevant by both reviewers were retrieved and reviewed. Two researchers independently assessed the full-text articles to confirm eligibility and reconcile any discrepancies. We additionally conducted a search for grey literature of evaluations of interventions to reduce contraceptive and abortion stigma by exploring the websites of key

For peer review only - http://bmjopen.bmj.com/site/about/guidelines.xhtml

organizations working in sexual and reproductive health and databases designed to extract unpublished literature, but located no relevant texts during this process.

Data charting process and analysis

We developed a data-charting form to extract relevant information to answer our scoping review question. One researcher classified and charted all studies according to study location, study aim, study design, type of contraceptive method(s), study population, type of stigma, and intervention approach (Supplementary Table 1). The second researcher reviewed to 20 percent of extractions of included articles to identify any discrepancies. Both researchers met to discuss, re-review articles and resolve any conflicts.

For the analysis of the included literature, we drew upon an existing stigma intervention framework outlined in a 2013 White Paper [20, 26] including the following categories: counselling approaches, education and training (information and skills-building), social marketing, protest and contact with affected groups. Although designed to address abortion stigma, the framework drew upon evidence from a range of health-stigmas and thus may also apply to contraceptive stigma. The interventions included in the four categories aim to address intrapersonal and interpersonal stigma (Table 1). The White Paper included an additional three categories for interventions designed to address stigma at the structural level: institutions, laws and policies, and the media [20]. We used this overall framework to organize the literature included in this scoping review. Finally, study quality was not assessed, as this is not a requirement for scoping reviews [22, 23].

For peer review only - http://bmjopen.bmj.com/site/about/guidelines.xhtml

Table 1. Categories of analysis

**Category**	**Definition**
Type of stigma [13, 15]	
Intrapersonal	Internalization of negative feelings, such as shame and guilt, associated with experience with abortion
Interpersonal	Direct or enacted forms of stigma, such as verbal harassment and/or physical assault based on association with abortion
Community	Social factors and attitudes and actions that cause individuals involved with abortion to lose status in their communities and social networks
Organizational/Institutional	Procedures and guidelines internal to organizations that perpetuate abortion stigma
Governmental/Structural	Educational, economic, legal, health policies and other regulation that reflects social norms that discredit abortion
Intervention approach [20]	
Counselling/peer support	Provide healthcare information, emotional support, and empowerment to people who experience stigma
Education/training/skills building	Provision of information about the stigmatized group and their concerns with the goal of reducing stigma
Contact	In-person interactions that can lead to reductions in prejudice between groups
Protest	An action that publicly calls attention to stigmatizing attitudes and/or behaviours that promote these attitudes

For peer review only - http://bmjopen.bmj.com/site/about/guidelines.xhtml

Social marketing	A strategy that uses marketing techniques to achieve behavioural and/or health-related goals
Media	Extended forms of contact that may involve fictional and nonfictional media or visualizations

For peer review only

For peer review only - http://bmjopen.bmj.com/site/about/guidelines.xhtml

Patient and public involvement

We did not engage with patients or the public during the development of this review.

Findings

Our initial search resulted in 16,096 records. After removing 4,192 duplicate articles, two researchers independently screened 11,904 titles and abstracts against the inclusion and exclusion criteria (Figure 1). The full texts of the 196 titles and abstracts were retrieved and reviewed. Of these, 178 were excluded (Figure 1). The remaining 18 texts were included in this review (Figure 1).

Characteristics of included articles

The 18 included studies were conducted in 10 countries: Cambodia (1), Colombia (1), India (1), Indonesia (1), Kenya (1), Mexico [1] (3), South Africa (1), Spain (1), and the United States of America (USA)(8). One study included unspecified countries in the Latin America and Caribbean region and Sub-Saharan Africa [27]. Of the 18 included studies, 11 reported on a quantitative evaluation, 6 reported on a qualitative evaluation, and 1 used mixed methods approaches. Fourteen of the studies focussed exclusively on abortion stigma, and two studies focussed on

[1] One study was conducted in Mexico City.

For peer review only - http://bmjopen.bmj.com/site/about/guidelines.xhtml

contraception stigma. The remaining two considered both. A majority aimed to address intrapersonal stigma. We found no interventions designed to address stigma at the structural level. Table 1 presents the full list of included studies.

Description of stigma reduction interventions

Of the 18 included papers, seven were categorized as education/training/skills building, five were categorized as counselling/peer support, three as contact, and three as media (Table 1). Each intervention is described by category below.

Education/training/skills building

Values clarification is a training intervention for addressing thoughts, feelings and attitudes towards issues that are emotive, such as contraception and abortion. The first article, published in 2015, was an evaluation of the effectiveness of the *Difficult Dialogues* strategies in reducing negative attitudes among midwives in South Africa [28]. The qualitative study suggested that the intervention had some influence on increasing participants' empathy for their patients [28]. The second article evaluated the TEACH (Teaching Everything About Contraceptive Health) program which aimed to dispel myths about contraception and abortion among obstetrican-gynocology residents in a faith-based hospital in the USA. The results of the evaluation suggested that the intervention had limited impact on reducing discriminatory attitudes towards patients seeking abortion services [29]. Finally, a recent evaluation of another values clarification workshop

For peer review only

conducted with residents in Catholic hopsitals in the USA found that, when presented with abortion related-scenarios, residents held less stigmatizing attitudes [30].

Researchers in Spain implemented an intervention for nursing students including a simulation of family planning counselling session. Using a quasi-experimental design, the evaluation determined that two weeks after the intervention, students' attitudes about contraceptive methods improved as measured by relevant validated scales [31].

An evaluation of an intervention conducted in India aimed to empower women to seek services and to normalize abortion and the provision of support by the community and family members of women who seek services. Via a quasi-experimental approach, a scale was used to assess attitudes towards abortion following the intervention. The results suggested that it increased perceived levels of social support for abortion within their families and self-efficacy around family planning and abortion. However, perceptions of support for abortion within communities were lower after the intervention [32].

The Family Planning Balance Counselling Strategy (FP-BCS) is an intervention designed to support the uptake of modern contraceptive methods. Researchers evaluated the effectiveness of the FP-BCS on attitudes, subjective norms and intentions to use modern contraception in Indonesia. An evaluation using a quasi-experimental design determined a significant increase in positive attitudes towards modern contraception among mothers as a result [33].

For peer review only - http://bmjopen.bmj.com/site/about/guidelines.xhtml

An evaluation of a school-based intervention assessed whether Comprehensive Sexuality Education reduced stigma related to abortion and contraceptive use in western Kenya. Among students ages 13-21 at follow-up, stigma scores for both contraception and abortion reduced significantly among boys and girls [7].

Researchers developed a narrative intervention that promoted cognitive restructuring and was designed to normalize the abortion experience using a animated 4-minute short-film. People seeking abortion viewed the animation in the waiting room before receiving care. A randomized controlled trial assessed changes to internalized stigma as a result of the intervention. At two-week follow-up there was no significant difference internalized stigma between intervention and control groups [34].

Counselling/peer support

The Story Circles Project intervention was implemented in Mexico and designed to reduce intrapersonal stigma among women who had experienced an abortion. The Story Circles Project provided a safe space for women to share their abortion stories, connect with other women and explore their abortion decision. The intervention also included information sessions to dispel myths and misinformation. The authors found that the intervention significantly reduced women's feelings of isolation helped reduce stigma [35].

Three evaluations of the Provider Share Workshop, an intervention designed for health workers involved in abortion care, were included in this review [36, 37, 38]. The workshop sessions offer

For peer review only - http://bmjopen.bmj.com/site/about/guidelines.xhtml

providers an opportunity to discuss and explore their experiences. The 2011 pilot study found that participants reported an “increased interpersonal connection” with the team following the workshop [36]. In 2014, the Provider Share Workshop was conducted among providers in the USA and effectiveness was evaluated using two validated scales. Although the evaluation could not show statistically significant results, the study identified a downward trend in abortion stigma overtime [37]. Finally, the Workshop was adapted and piloted in two countries in Sub-Saharan Africa and Latin America. The evaluation found decreases in overall abortion stigma, but specifically in disclosure management, internalized stigma and judgement [38].

A qualitative assessment of the role of counselling within legal abortion services offered in non-governmental organizations in Mexico City and Colombia in addressing women’s concerns and fears prior to receiving abortion care found that women experienced relief, and less guilt and internalised stigma after counselling [38].

Contact

In 2018, researchers assessed the effectiveness of an intervention designed to reduce interpersonal abortion stigma manifesting as secrecy among women who have experienced abortion and negative reactions from women who learn about the abortion experiences of others. The intervention used the safe spaces of exisiting book clubs to assess whether women would self-disclose their abortion experiences in a positive face-to-face interaction. Book clubs were recruited to read and discuss the book *Choice: True Stories of Birth, Contraception, Infertility, Adoption, Single Parenthood and Abortion*. During the book club intervention, a majority of women

For peer review only - http://bmjopen.bmj.com/site/about/guidelines.xhtml

with abortion histories disclosed their experience and the book “warmed” participants feelings about abortion over time [39].

The online platform, Lightbulbs (*Focos*, in Spanish), was designed in Mexico to provide a safe space for women to tell their abortion stories and to increase abortion visibility. In discourse analysis of the text, researchers found that most of the stories were positive and described as a “cathartic” experience [40].

Media

In 2016, researchers examined in the impact of the viewing of the documentary film, *After Tiller*, in reducing third trimester abortion stigma. Viewing the film appeared to challenge myths and misperceptions about abortion. However, the authors found that the medium was limited in its ability to generate new belief systems around abortion [41].

The results of an online study of viewers on an episode of the USA medical television program *Grey’s Anatomy* which featured an abortion storyline titled “Papa Don’t Preach”. The findings suggested that the program did not increase viewers willingness to support a friend seeking an abortion, whether medically necessary or induced [42].

Discussion

For peer review only - http://bmjopen.bmj.com/site/about/guidelines.xhtml

Despite calls for the development of interventions focussed on reducing reproductive health stigmas, and specifically, contraceptive and abortion stigma, there remains significant gaps in the literature [4, 16, 18, 19]. Most articles identified were reports of evaluations of abortion stigma interventions taking place in the USA. A handful of articles focussed on Latin America, two in Sub-Saharan Africa, one in Asia and one in Europe demonstrating limitations of understanding of available interventions in specific regions and contexts.

The published interventions largely focussed on addressing intrapersonal and interpersonal stigma. The most common intervention approach identified was education, training and skills building. The least common was the contact approach. There is a significant gap in the availability of interventions to reduce contraceptive and abortion stigma at the structural level and using approaches involving institutions, and laws and policies.

There exists substantial theory about intervention approaches that should have an effect on reducing contraception and abortion stigma [1, 15, 17, 18]. Several rigoursly validated scales are available for measuring changes in contraception and abortion stigma. Program developers and evaluators should design and test interventions based on theory and measure their effectiveness using existing validated scales. Future research may synthesize the existing evidence to understand the effectiveness of specific intervention approaches.

The methodology of this scoping review has some limitations. We limited our search to studies that were published in English; this may have inadvertently excluded key articles written in other

For peer review only

languages. We focused on studies that had an element of evaluation. This meant that we excluded descriptions of activities taking place to reduce contraception and abortion stigma without efforts to measure effectiveness, acceptability or implementation. This methodological choice may have limited our engagement with interventions currently in use, but not evaluated. It is also possible that evaluators have not published the results of studies of ineffective or inconclusive interventions.

In the design of interventions, stigma continues to be largely conceptualised as an attribute and interventions to mitigate stigma targeted to manifestations at the individual level. Any interventions aligning with this concept may inadvertently (re)generate stigma and minimize the impact of mitgation efforts. Further attention in the development of interventions targeted to the social processes that underpin stigma may have a more profound impact on attitudes and behaviours across populations [43]. Furthermore, efforts to reduce contraceptive and abortion stigma could draw upon a broader stigma framework and benefit from shared concepts, tools, indicators and outcomes [44]. Scoping reviews are pragamatic precursors to systematic reviews where the scope and characteristics of an area of investigation is unclear [22]. This results of this scoping review can inform efforts to develop robust systematic reviews to assess the quality and effectiveness of approaches to reduce contraceptive and abortion stigma.

Acknowledgements

The authors would like to acknowledge the input of Kavita Kothari, World Health Organization Librarian, Geneva, Switzerland for her suggestions and support in refining the search strategy.

For peer review only

Disclaimer

The views expressed in this article are those of the authors and do not necessarily represent the views of, and should not be attributed to, the World Health Organization.

Funding

The UNDP-UNFPA-UNICEF-WHO-World Bank Special Programme of Research, Development and Research Training in Human Reproduction (HRP), a co-sponsored program executed by the World Health Organization (WHO), funded this work.

Competing interests None declared.

Patient consent for publication Not required

Provenance and peer review Not commissioned; externally peer reviewed.

Data availability statement All data relevant to the study are included in the article or uploaded as supplementary information.

Ethics approval An ethics statement is not applicable because this study was based exclusively on published literature.

For peer review only

Contributorship statement AMS and URL conceptualized and conducted the review, including assessing literature and data synthesis. AMS prepared the manuscript with intellectual contributions from URL. Both authors approved the final manuscript.

For peer review only

References

1. Scrambler G, Heijnders M and van Brakel WH on behalf of ICRAAS. (2006) Understanding and tackling health-related stigma. Psychology, Health & Medicine, 11:3, 269-270, DOI: 10.1080/13548500600594908

2. Hatzenbuehler ML, Phelan JC and Link BG. (2013) Stigma as a fundamental cause of population health inequalities. American Journal of Public Health, 103(5): 813-21

3. Ohlsson-Nevo E and Karlsson J. (2018) Impact of health-related stigma on psychosocial functioning in the general population: Construct validity and Swedish reference data for the Stigma-related Social Problems scale (SSP). Research in Nursing and Health, 42: 72-82.

4. Cook RJ and Dicken BM (2014) Reducing stigma in reproductive health. International Journal of Gynecology and Obstetrics, 125: 89-92.

5. Makenzius M, McKinney G, Oguttu M. et al. Stigma related to contraceptive use and abortion in Kenya: scale development and validation. Reprod Health 16, 136 (2019). https://doi.org/10.1186/s12978-019-0799-1

6. Ostrach B (2012) The tangled web of reproductive morbidity risk: Abortion stigma, safety and legality. Frontiers in Women's Health 1 (2)

7. Rehnström Loi U. (2020) Abortion, contraception and associated social stigma – consequences and solutions in a low-resource setting in Western Kenya. PhD Thesis. Karolinska Institutet, Stockholm, Sweden.

8. Blodgett M, Weidert K, Nieto-Andrade B, and Prata N. (2018) Do perceived contraception attitudes influence abortion stigma? Evidence from Luanda, Angola. Social Science and Medicine - Population Health; 5: 38-47.

For peer review only - http://bmjopen.bmj.com/site/about/guidelines.xhtml

9. Farmer DB, Berman L, Ryan G, Habumugisha L, Basinga P, Nutt C, Kamali F, Ngizwenayo E, St. Fluer J, Niyigena P, Ngabo F, Farmer PE and Rich ML. (2015) Motivations and contraints to family planning: A qualitative study in Rwanda's Southern Kayonza District. Global Health: Science and Practice, 3(2):242-254; https://doi.org/10.9745/GHSP-D-14-00198

10. Bearak JM, Popinchalk A, Beavin C, Ganatra B, Moller AB, Tunçalp Ö, and Alkema L. (2022) Country-specific estimates of unintended pregnancy and abortion incidence: a global comparative analysis of levels in 2015–2019. BMJ Global Health;7:e007151. doi:10.1136/ bmjgh-2021-007151

11. Hanschmidt F, Linde K, Hilbert A, Riedel-Heller SG and Kersting A (2016) Abortion stigma: A systematic review. Perspectives on Sexual and Reproductive Health, 48(4): doi: 10.1363/48e8516

12. Moore B, Poss C, Coast E, Lattof SR, van der Meulen Rodgers Y (2021) The economics of abortion and its links with stigma: A secondary analysis from a scoping review on the economics of abortion. PLoS ONE 16(2): e0246238. https://doi.org/10.1371/journal.pone.0246238

13. Kumar A, Hesseni L and Mitchell EMH (2009) Conceptualising abortion stigma. Culture, Health and Sexuality, Aug;11(6):625-39. doi: 10.1080/13691050902842741.

14. Norris A, Bessett D, Steinberg JR, Kavanaugh ML, De Zordo S, and Becker D. (2011) Abortion stigma: A reconceptualization of constituents, causes, and consequences. Women's Health Issues, doi:10.1016/j.whi.2011.02.010

15. Heijinders M and van Der Meij (2006) The fight against stigma: an overview of stigma-reduction strategies and interventions. Psychol Health Med, Aug;11(3):353-63. doi: 10.1080/13548500600595327

For peer review only - http://bmjopen.bmj.com/site/about/guidelines.xhtml

16. Rehnström Loi U, Otieno B, Oguttu M, Gemzell-Danielsson K, Klingberg-Allvin M, Faxelid E and Makenzius M (2019) Abortion and contraceptive use stigma: a cross-sectional study of attitudes and beliefs in secondary school students in western Kenya. Sexual and Reproductive Health Matters, 27:3, 20-31, DOI: 10.1080/26410397.2019.1652028

17. Hussein J and Ferguson L (2019) Eliminating stigma and discrimination in sexual and reproductive health care: a public health imperative, Sexual and Reproductive Health Matters, 27:3, 1-5, DOI: 10.1080/26410397.2019.1697103

18. Sorhaindo A and Lavelanet AF. Why does abortion stigma matter? A scoping review and conceptual framework describing the role of stigma in shaping abortion quality of care. *Social Science and Medicine*, https://doi.org/10.1016/j.socscimed.2022.115271.

19. Rao D, Elshafei A, Nguyen M, Hatzenbuehler ML, Frey S and Go VF. (2019) A systematic review of multi-level stigma interventions: state of the science and future directions. 17:41 https://doi.org/10.1186/s12916-018-1244-y

20. Cockrill K, Herold S, Blanchard K, Grossman D, Upadhyay U, Baum S. (2013a). Addressing Abortion Stigma Through Service Delivery: A White Paper. Retrieved from Ibis Reproductive Health: https://www.ibisreproductivehealth.org/publications/addressing-abortion-stigma-through-service-delivery-white-paper.

21. Armstrong R, Hall BJ Doyle J and Waters E. (2011) "Scoping the scope" of a cochrane review. Journal of Public Health, 33(1): 147-150.

22. Munn Z, Peters MDJ, Stern C, Tufanaru, McArthur A and Aromataris E. (2018) Systematic review or scoping review? Guidance for authors when choosing between a systematic or scoping review approach. BMC Medical Research Methodology, 18:143, https://doi.org/10.1186/s12874-018-0611-x

For peer review only

23. Arksey, H and O'Malley, L (2005) Scoping studies: towards a methodological framework. International Journal of Social Research Methodology, 19-32.

24. Levac D, Colquhoun H, O'Brien KK. (2010) Scoping studies: advancing the methodology. Implement Sci, 5:69.

25. Moher D, Shamseer L, Clarke M, Ghersi D, Liberati A, Petticrew M et al. (2015) Preferred reporting items for systematic review and meta-analysis protocols (PRISMA-P) 2015 statement. Syst Rev, 4: 1 https://doi.org/10.1186/2046-4053-4-1

26. Brown, L., Macintyre, K., & Trujillo, L. (2003). Interventions to reduce HIV/AIDS stigma: What have we learned? AIDS Education and Prevention, 15(1), 49-69.

27. Mosley EA, Martin L, Seewald M, Hassinger J, Blanchard K, Baum SE, Santana D, Echeverri L, Garrett J, Njunguru J and Harris L (2020). Addressing abortion provider stigma: A pilot implementation of the Providers Share Workshop in sub-Saharan Africa and Latin America. International Perspectives on Sexual and Reproductive Health, 46: 35-50.

28. Mpeli MR and Botma Y (2015) Abortion-related services: Values clarification through 'Diffcult Dialogues' strategies. Education, Citizenship and Social Justice, 10(3): 278-288

29. Guiahi M, Cortland C, Graham MJ, Heraty S, Lukens M, Trester M, Summers S, Kenton K (2011) Addressing OB/GYN family planning educational objectives at a faith-based institution using the TEACH program. Contraception, 83: 367-372.

30. Guiahi M, Wilson C, Claymore E, Simonson K, Steinauer J. (2021) Influence of a values clarification workshop on residents training at Catholic Hospital programs. Contraception: X 3 100054

31. Sanz-Martos S, López-Medina IM, Álvarez-Garcia C, Álvarez-Nieto C. (2021) Educational program on sexuality and contraceptive methods in nursing degree students. Nurse Education Today, 107: 105114

For peer review only - http://bmjopen.bmj.com/site/about/guidelines.xhtml

32. Banerjee SK, Andersen KL, Warvadekar J and Pearson E. (2013) Intervention to improve knowledge and perceptions abortion abortion in Bihar and Jharkhand, India. International Perspectives in Sexual and Reproductive Health, 39(3): 142-151, doi:10.1363/3914213

33. Palinggi RS, Moedjiono AI, Suarayasa K, Masni, Seweg A, Amqam H, Nur R amd Syam A (2021) The effect of balanced counseling strategy family planning against attitude, subjective norm, and intentions on the use of modern contraception behavior in the Singgani Public Health Center work area of Palu city. Gac Sanit

34. Sackeim MG, Lee SY, Newton S, Lauderdale D, Gilliam M (2022). Effect of a narrative intervention on individual-level abortion stigma: a randomised controlled trial. BMJ Sexual and Reproductive Health; 48:47–53.

35. Belfrage M, Ortíz Ramírez O and Sorhaindo A. (2019) Story Circles and abortion stigma in Mexico: a mixed-methods evaluation of a new intervention for reducing individual level abortion stigma, Culture, Health & Sexuality, DOI: 10.1080/13691058.2019.1577493

36. Harris LH, Debbink M, Martin L, and Hassinger J (2011) Dynamics of stigma in abortion work: Findings from a pilot of the Providers Share Workshop. Social Science and Medicine, 73: 1062-1070.

37. Martin LA, Debbink M, Hassinger J, Youatt E and Harris LH. (2014) Abortion providers, stigma and professional quality of life. Contraception, 90: 581-587.

38. Keefe-Oates B, Makleff S, Sa E, Forero LJ, Mendoza D, Olaya MA. (2019) Experiences with abortion counselling in Mexico City and Colombia: addressing women's fears and concerns. Culture, Health and Sexuality, 4.

39. Cockrill K and Biggs A. (2018) Can stories reduce abortion stigma? Findings from a longitudinal cohort study. Culture, Health and Sexuality, 3.

For peer review only - http://bmjopen.bmj.com/site/about/guidelines.xhtml

40. Belfrage M, Didier E and Vázquez-Quesada L (2021): Voicing Abortion Experiences to Reduce Stigma: Lessons from an Online Storytelling Platform in Mexico, Women's Reproductive Health, DOI: 10.1080/23293691.2021.2016145

41. Sisson G and Kimport K (2016) After After Tiller: the impact of a documentary film on understandings of third-trimester abortion. Culture, Health and Sexuality: 6.

42. Sisson G, Walter N, Harold S and Brooks JJ. (2021) Prime-time abortion on Grey's Anatomy: What do US viewers learn from fictional portrayals of abortion on television?

43. Millar E. (2020) Abortion stigma is a social process. Women's Studies International Forum, 78: 102328

44. Stangl AL, Earnshaw VA, Logie CH, van Brakel W, Simbayi LC, Barré I and Dovidio JF. (2019) The Health Stigma and Discrimination Framework: a global, crosscutting framework to inform research, intervention development, and policy on health-related stigmas. BMC Medicine, 17:31

For peer review only

For peer review only - http://bmjopen.bmj.com/site/about/guidelines.xhtml

Figure 1. Scoping review PRISMA flowchart

For peer review only

For peer review only - http://bmjopen.bmj.com/site/about/guidelines.xhtml

Figure 1. Scoping review PRISMA flowchart

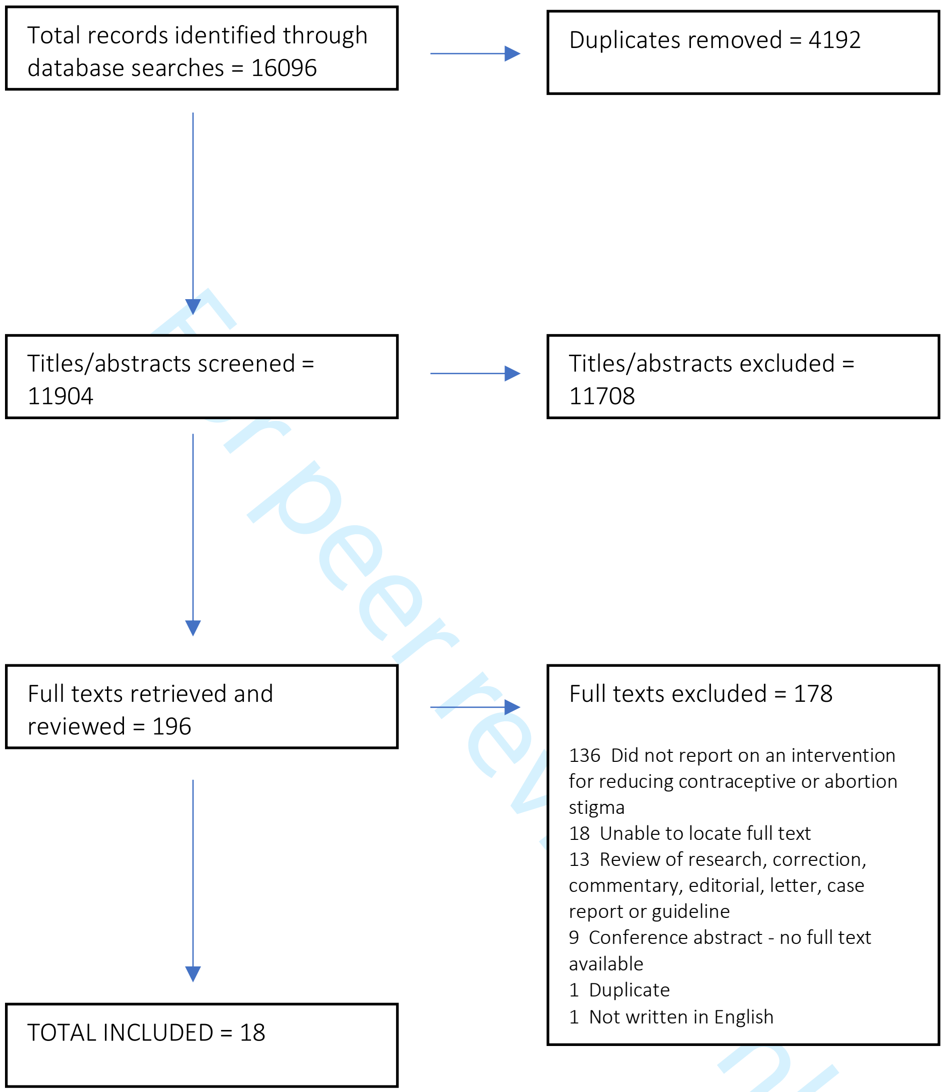

For peer review only

Supplemental material

Supplementary Table 1. Characteristics of included studies (n=18)

Author	Year of publication	Country	Scope	Evaluation design	Focus	Type of stigma	Intervention approach
Banerjee	2013	India	To evaluate the effectiveness of a behavior change communication intervention to improve women's knowiedge about India's abortion law and their perceptions about abortion	Quasi-experimental	Abortion	Community	Education/training/skills building
Belfarge	2020	Mexico	The evaluation measured whether participation in the intervention reduced women's experience of individual level stigma one month after having participated and explored women's expressed experiences of the intervention and their perceptions of changes in stigmatising feelings after the intervention.	Mixed methods	Abortion	Intrapersonal	Counselling/peer support
Belfarge	2021	Mexico	To understand more about who tells their stories online, how they represent their abortion experience, and what lessons this leaves us as feminist activists seeking to advance sexual and reproductive rights through online storytelling	Qualitative	Abortion	Intrapersonal	Contact
Cockrill	2018	USA	To measure changes in women's attitudes toward abortion and their willingness to disclose their own abortion experiences with one another	Quasi-experimental	Abortion	Interpersonal	Contact

For peer review only

			following a book group discussion in which participants discussed a previously chosen book.				
Guiahi	2011	USA	To evaluate if a values clarification workshop conducted at Catholic hospital training programs influenced obstetrics and gynecology residents' abortion attitudes.	Quasi-experimental	Abortion	Interpersonal	Education/training/skills building
Guiahi	2021	USA	To understand whether a values clarification workshop influences abortion attitudes of obgyn residents at U.S. training programs that are based in Catholic hospitals and lack abortion training.	Quasi-experimental	Abortion	Interpersonal	Education/training/skills building
Harris	2011	USA	To present findings from a qualitative pilot study of the Providers Share Workshop, a six-session workshop in which abortion providers (broadly defined to include anyone with direct daily involvement in abortion care), meet to explore their experiences, guided by an experienced facilitator	Qualitative	Abortion	Intrapersonal	Counselling/peer support
Keefe-Oates	2020	Colombia and Mexico City	To develop a greater understanding of their clients' concerns prior to abortion care,and the role that counselling can play in addressing these concerns.	Qualitative	Abortion	Intrapersonal	Counselling/peer support
Martin	2014	USA	The Providers Share Workshop (PSW) provides abortion providers safe space to discuss their work experiences. Our	Quasi-experimental	Abortion	Intrapersonal	Counselling/peer support

For peer review only

			objectives were to assess changes in abortion stigma over time and explore how stigma is related to aspects of professional quality of life, including compassion satisfaction, burnout and compassion fatigue for providers participating in the workshops.				
Mosley	2020	Sub-Saharan Africa and Latin American and the Caribbean*	To adapt the Providers Share Workshop content, structure and evaluation tools for this pilot study in Sub-Saharan Africa and Latin America.	Cohort	Abortion	Intrapersonal	Counselling/peer support
Mpeli	2015	South Africa	To report on the value clarification process and the views of preregistration midwifery students regarding abortion-related services before and after implementing the value clarification process.	Qualitative	Abortion	Intrapersonal	Education/training/skills building
Palinggi	2021	Indonesia	To analyze the influence of the Balanced Counseling Strategy (FP-BCS) on attitudes, subjective norms, and intentions on modern contraception in the working area of Singgani Health Center, Palu, Indonesia	Quasi-experimental	Contraception	Community	Education/training/skills building
Rehnström Loi	2020	Kenya	To measure attitudes towards abortion and contraceptive use, and to evaluate a stigma-reduction intervention among secondary school students.	Quasi-experimental	Both	Interpersonal	Education/training/skills building

For peer review only

Sackeim	2022	USA	To evaluate the effect of a narrative intervention on individual-level abortion stigma in patients undergoing abortion.	Randomized Controlled Trial	Abortion	Intrapersonal	Contact
San-Martos	2021	Spain	This study aimed to evaluate the effect of a peer-educational intervention to improve knowledge level and attitudes regarding contraceptive methods in university students.	Quasi-experimental	Contraception	Community	Education/training/skills building
Sisson	2016	USA	To determine whether a documentary film can impact stigmatizing attaitudes against 3rd trimester abortion.	Qualitative	Abortion	Community	Media
Sisson	2021	USA	To examine the impact of an abortion storyline from the TV show Grey's Anatomy on USA-based viewers.	Quasi-experimental	Abortion	Community	Media
Smith	2021	Cambodia	The aim of this study was to describe the development of an intervention to support the reproductive health of garment factory workers in Cambodia	Qualitative	Both	Community	Media

*Only regions were report

For peer review only

Appendix 1. Preferred Reporting Items for Systematic reviews and Meta-Analyses extension for Scoping Reviews (PRISMA-ScR) Checklist

SECTION	ITEM	PRISMA-ScR CHECKLIST ITEM	REPORTED ON PAGE #
TITLE			
Title	1	Identify the report as a scoping review.	1
ABSTRACT			
Structured summary	2	Provide a structured summary that includes (as applicable): background, objectives, eligibility criteria, sources of evidence, charting methods, results, and conclusions that relate to the review questions and objectives.	2
INTRODUCTION			
Rationale	3	Describe the rationale for the review in the context of what is already known. Explain why the review questions/objectives lend themselves to a scoping review approach.	4
Objectives	4	Provide an explicit statement of the questions and objectives being addressed with reference to their key elements (e.g., population or participants, concepts, and context) or other relevant key elements used to conceptualize the review questions and/or objectives.	4
METHODS			
Protocol and registration	5	Indicate whether a review protocol exists; state if and where it can be accessed (e.g., a Web address); and if available, provide registration information, including the registration number.	N/A
Eligibility criteria	6	Specify characteristics of the sources of evidence used as eligibility criteria (e.g., years considered, language, and publication status), and provide a rationale.	5
Information sources*	7	Describe all information sources in the search (e.g., databases with dates of coverage and contact with authors to identify additional sources), as well as the date the most recent search was executed.	5
Search	8	Present the full electronic search strategy for at least 1 database, including any limits used, such that it could be repeated.	26
Selection of sources of evidence†	9	State the process for selecting sources of evidence (i.e., screening and eligibility) included in the scoping review.	5
Data charting process‡	10	Describe the methods of charting data from the included sources of evidence (e.g., calibrated forms or forms that have been tested by the team before their use, and whether data charting was done independently or in	5-6

For peer review only

SECTION	ITEM	PRISMA-ScR CHECKLIST ITEM	REPORTED ON PAGE #
		duplicate) and any processes for obtaining and confirming data from investigators.	
Data items	11	List and define all variables for which data were sought and any assumptions and simplifications made.	22
Critical appraisal of individual sources of evidence§	12	If done, provide a rationale for conducting a critical appraisal of included sources of evidence; describe the methods used and how this information was used in any data synthesis (if appropriate).	N/A
Synthesis of results	13	Describe the methods of handling and summarizing the data that were charted.	6
RESULTS			
Selection of sources of evidence	14	Give numbers of sources of evidence screened, assessed for eligibility, and included in the review, with reasons for exclusions at each stage, ideally using a flow diagram.	15
Characteristics of sources of evidence	15	For each source of evidence, present characteristics for which data were charted and provide the citations.	16-21
Critical appraisal within sources of evidence	16	If done, present data on critical appraisal of included sources of evidence (see item 12).	N/A
Results of individual sources of evidence	17	For each included source of evidence, present the relevant data that were charted that relate to the review questions and objectives.	Click here to enter text.
Synthesis of results	18	Summarize and/or present the charting results as they relate to the review questions and objectives.	7-9
DISCUSSION			
Summary of evidence	19	Summarize the main results (including an overview of concepts, themes, and types of evidence available), link to the review questions and objectives, and consider the relevance to key groups.	9-10
Limitations	20	Discuss the limitations of the scoping review process.	10
Conclusions	21	Provide a general interpretation of the results with respect to the review questions and objectives, as well as potential implications and/or next steps.	9-10
FUNDING			
Funding	22	Describe sources of funding for the included sources of evidence, as well as sources of funding for the scoping review. Describe the role of the funders of the scoping review.	10

JBI = Joanna Briggs Institute; PRISMA-ScR = Preferred Reporting Items for Systematic reviews and Meta-Analyses extension for Scoping Reviews.
* Where *sources of evidence* (see second footnote) are compiled from, such as bibliographic databases, social media platforms, and Web sites.
† A more inclusive/heterogeneous term used to account for the different types of evidence or data sources (e.g., quantitative and/or qualitative research, expert opinion, and policy

For peer review only

documents) that may be eligible in a scoping review as opposed to only studies. This is not to be confused with *information sources* (see first footnote).
‡ The frameworks by Arksey and O'Malley (6) and Levac and colleagues (7) and the JBI guidance (4, 5) refer to the process of data extraction in a scoping review as data charting.
§ The process of systematically examining research evidence to assess its validity, results, and relevance before using it to inform a decision. This term is used for items 12 and 19 instead of "risk of bias" (which is more applicable to systematic reviews of interventions) to include and acknowledge the various sources of evidence that may be used in a scoping review (e.g., quantitative and/or qualitative research, expert opinion, and policy document).

From: Tricco AC, Lillie E, Zarin W, O'Brien KK, Colquhoun H, Levac D, et al. PRISMA Extension for Scoping Reviews (PRISMAScR): Checklist and Explanation. Ann Intern Med. 2018;169:467–473. doi: 10.7326/M18-0850.

For peer review only

Appendix 2. Example search strategy

	#	Searches
Stigma		Social stigma* OR shame* OR guilt* OR discriminat* OR Prejudice* OR judgement OR stereotype*OR blame OR isolate* OR moral* OR harass* OR insult* OR labeling OR social-accept* OR social-approv* OR social-percep*OR treatment-barrier*
Abortion or contraception		abortion, induced OR Abortion, Criminal OR Abortion, Septic OR Abortion Applicants OR abortion, missed OR abortion, legal OR abortion, spontaneous OR abortion* OR pregnancy-terminat* OR post-abortion*OR Postconception Fertility Control OR Embryotomies OR Embryotomy OR Dilatation and curettage OR Vacuum Curettage OR Surgical abortion OR Manual vacuum aspiration OR (vacuum AND aspiration) OR Curettage OR Surgical termination of pregnancy OR Dilatation and evacuation OR Dilation and evacuation OR Suction aspiration OR Aspiration abortion OR Suction curettage OR Vacuum curettage OR Contraception OR Contraception Behavior OR Condom OR Condoms OR Diaphragm OR Diaphragms OR Cervical Cap OR Vaginal-sponge OR Cervical-Caps OR Vaginal-sponges OR Birth-Control OR Contraceptive-Methods OR Contraceptive-Method OR Female Contraception OR Female Contraceptions OR Male Contraception OR Male Contraceptions OR Inhibition-of-Fertilization OR Fertilization-Inhibition OR Fertility-Control OR Birth-Control OR Vaginal-Barrier OR Hormonal-Contracept* OR anticonception OR antifertility OR conception-control OR family planning OR birth control OR birth-prevention OR pregnancy-prevent*
Program evaluation-related		intervention OR program OR activity OR action

For peer review only

Research Checklist

Appendix 1. Preferred Reporting Items for Systematic reviews and Meta-Analyses extension for Scoping Reviews (PRISMA-ScR) Checklist

SECTION	ITEM	PRISMA-ScR CHECKLIST ITEM	REPORTED ON PAGE #
**TITLE**			
Title	1	Identify the report as a scoping review.	1
**ABSTRACT**			
Structured summary	2	Provide a structured summary that includes (as applicable): background, objectives, eligibility criteria, sources of evidence, charting methods, results, and conclusions that relate to the review questions and objectives.	2
**INTRODUCTION**			
Rationale	3	Describe the rationale for the review in the context of what is already known. Explain why the review questions/objectives lend themselves to a scoping review approach.	4
Objectives	4	Provide an explicit statement of the questions and objectives being addressed with reference to their key elements (e.g., population or participants, concepts, and context) or other relevant key elements used to conceptualize the review questions and/or objectives.	4
**METHODS**			
Protocol and registration	5	Indicate whether a review protocol exists; state if and where it can be accessed (e.g., a Web address); and if available, provide registration information, including the registration number.	N/A

For peer review only

SECTION	ITEM	PRISMA-ScR CHECKLIST ITEM	REPORTED ON PAGE #
Eligibility criteria	6	Specify characteristics of the sources of evidence used as eligibility criteria (e.g., years considered, language, and publication status), and provide a rationale.	5
Information sources*	7	Describe all information sources in the search (e.g., databases with dates of coverage and contact with authors to identify additional sources), as well as the date the most recent search was executed.	5
Search	8	Present the full electronic search strategy for at least 1 database, including any limits used, such that it could be repeated.	26
Selection of sources of evidence†	9	State the process for selecting sources of evidence (i.e., screening and eligibility) included in the scoping review.	5
Data charting process‡	10	Describe the methods of charting data from the included sources of evidence (e.g., calibrated forms or forms that have been tested by the team before their use, and whether data charting was done independently or in duplicate) and any processes for obtaining and confirming data from investigators.	5-6
Data items	11	List and define all variables for which data were sought and any assumptions and simplifications made.	22
Critical appraisal of individual sources of evidence§	12	If done, provide a rationale for conducting a critical appraisal of included sources of evidence; describe the methods used and how this information was used in any data synthesis (if appropriate).	N/A
Synthesis of results	13	Describe the methods of handling and summarizing the data that were charted.	6

For peer review only

SECTION	ITEM	PRISMA-ScR CHECKLIST ITEM	REPORTED ON PAGE #
**RESULTS**			
Selection of sources of evidence	14	Give numbers of sources of evidence screened, assessed for eligibility, and included in the review, with reasons for exclusions at each stage, ideally using a flow diagram.	15
Characteristics of sources of evidence	15	For each source of evidence, present characteristics for which data were charted and provide the citations.	16-21
Critical appraisal within sources of evidence	16	If done, present data on critical appraisal of included sources of evidence (see item 12).	N/A
Results of individual sources of evidence	17	For each included source of evidence, present the relevant data that were charted that relate to the review questions and objectives.	Click here to enter text.
Synthesis of results	18	Summarize and/or present the charting results as they relate to the review questions and objectives.	7-9
**DISCUSSION**			
Summary of evidence	19	Summarize the main results (including an overview of concepts, themes, and types of evidence available), link to the review questions and objectives, and consider the relevance to key groups.	9-10
Limitations	20	Discuss the limitations of the scoping review process.	10
Conclusions	21	Provide a general interpretation of the results with respect to the review questions and objectives, as well as potential implications and/or next steps.	9-10
**FUNDING**			

For peer review only

SECTION	ITEM	PRISMA-ScR CHECKLIST ITEM	REPORTED ON PAGE #
Funding	22	Describe sources of funding for the included sources of evidence, as well as sources of funding for the scoping review. Describe the role of the funders of the scoping review.	10

JBI = Joanna Briggs Institute; PRISMA-ScR = Preferred Reporting Items for Systematic reviews and Meta-Analyses extension for Scoping Reviews.
* Where *sources of evidence* (see second footnote) are compiled from, such as bibliographic databases, social media platforms, and Web sites.
† A more inclusive/heterogeneous term used to account for the different types of evidence or data sources (e.g., quantitative and/or qualitative research, expert opinion, and policy documents) that may be eligible in a scoping review as opposed to only studies. This is not to be confused with *information sources* (see first footnote).
‡ The frameworks by Arksey and O'Malley (6) and Levac and colleagues (7) and the JBI guidance (4, 5) refer to the process of data extraction in a scoping review as data charting.
§ The process of systematically examining research evidence to assess its validity, results, and relevance before using it to inform a decision. This term is used for items 12 and 19 instead of "risk of bias" (which is more applicable to systematic reviews of interventions) to include and acknowledge the various sources of evidence that may be used in a scoping review (e.g., quantitative and/or qualitative research, expert opinion, and policy document).

From: Tricco AC, Lillie E, Zarin W, O'Brien KK, Colquhoun H, Levac D, et al. PRISMA Extension for Scoping Reviews (PRISMAScR): Checklist and Explanation. Ann Intern Med. 2018;169:467–473. doi: 10.7326/M18-0850.

For peer review only

For peer review only

BMJ Open

Interventions to reduce stigma related to contraception and abortion: a scoping review

Journal:	*BMJ Open*
Manuscript ID	bmjopen-2022-063870.R2
Article Type:	Original research
Date Submitted by the Author:	12-Oct-2022
Complete List of Authors:	Sorhaindo, Annik; World Health Organization, Sexual and Reproductive Health and Research<br>Rehnstrom Loi, Ulrika; World Health Organization, Sexual and Reproductive Health and Research
**Primary Subject Heading**:	Public health
Secondary Subject Heading:	Reproductive medicine
Keywords:	PUBLIC HEALTH, REPRODUCTIVE MEDICINE, MEDICAL ETHICS

SCHOLARONE™
Manuscripts

I, the Submitting Author has the right to grant and does grant on behalf of all authors of the Work (as defined in the below author licence), an exclusive licence and/or a non-exclusive licence for contributions from authors who are: i) UK Crown employees; ii) where BMJ has agreed a CC-BY licence shall apply, and/or iii) in accordance with the terms applicable for US Federal Government officers or employees acting as part of their official duties; on a worldwide, perpetual, irrevocable, royalty-free basis to BMJ Publishing Group Ltd ("BMJ") its licensees and where the relevant Journal is co-owned by BMJ to the co-owners of the Journal, to publish the Work in this journal and any other BMJ products and to exploit all rights, as set out in our licence.

The Submitting Author accepts and understands that any supply made under these terms is made by BMJ to the Submitting Author unless you are acting as an employee on behalf of your employer or a postgraduate student of an affiliated institution which is paying any applicable article publishing charge ("APC") for Open Access articles. Where the Submitting Author wishes to make the Work available on an Open Access basis (and intends to pay the relevant APC), the terms of reuse of such Open Access shall be governed by a Creative Commons licence – details of these licences and which Creative Commons licence will apply to this Work are set out in our licence referred to above.

Other than as permitted in any relevant BMJ Author's Self Archiving Policies, I confirm this Work has not been accepted for publication elsewhere, is not being considered for publication elsewhere and does not duplicate material already published. I confirm all authors consent to publication of this Work and authorise the granting of this licence.

For peer review only

Title

Interventions to reduce stigma related to contraception and abortion: a scoping review

Authors

Annik Mahalia Sorhaindo, MSc PhD[1], Ulrika Rehnström Loi, MSc PhD[1*]

*Corresponding author: Ulrika Rehnström Loi, 20 Avenue Appia, 1211 Geneva, Switzerland, e-mail: rehnstromu@who.int

1. UNDP-UNFPA-UNICEF-WHO-World Bank Special Programme of Research, Development and Research Training in Human Reproduction (HRP), Department of Sexual and Reproductive Health and Research, World Health Organization, 20 Avenue Appia, 1211 Geneva, Switzerland

Key words (9): abortion stigma, contraceptive use stigma, values clarification, intervention, evaluation

For peer review only - http://bmjopen.bmj.com/site/about/guidelines.xhtml
For peer review only

Word count: 4659/5000 (including summary box, references, tables and figures)

Tables: 2 Figures: 1

For peer review only

For peer review only - http://bmjopen.bmj.com/site/about/guidelines.xhtml

Abstract

Objectives:

We conducted a scoping review to identify the types, volume and characteristics of available evidence and analyze gaps in the knowledge base for evaluated interventions to reduce contraception and abortion stigma.

Design

We conducted a search of five electronic databases to identify articles published between January 2000 and January 2022, and explored the websites of relevant organizations and grey literature databases for unpublished and non-commercial reports. Articles were assessed for eligibility, and data were extracted.

Data sources

We searched MEDLINE, PubMED, EMBASE, Web of Science and PsychINFO.

Eligibility criteria

Articles included were: (1) published between January 2000 and January 2022, (2) written in English, (3) reports of the evaluation of an intervention designed to reduce contraceptive and/or abortion stigma (4) used any type of study design, and (5) conducted in any country context.

Data extraction and synthesis

For peer review only - http://bmjopen.bmj.com/site/about/guidelines.xhtml

Included studies were charted according to study location, study aim, study design, type of contraceptive method(s), study population, type of stigma, and intervention approach.

Results

Some 18 articles were included in the final analysis (11 quantitative, 6 qualitative, and 1 mixed methods). Fourteen of the studies focussed exclusively on abortion stigma, and 2 studies focussed on contraception stigma only. Two considered both. A majority of studies aimed to address intrapersonal stigma. We found no interventions designed to address stigma at the structural level. In terms of intervention approaches, seven were categorized as education/training/skills building, five as counselling/peer support, three as contact, and three as media.

Conclusion

There is a dearth of evaluations of interventions to reduce contraception and abortion stigma. Investment in implementation science is necessary to develop the evidence base and inform the development of effective interventions, and use existing stigma scales to evaluate effectiveness. This scoping review can serve as a precursor to systematic reviews assessing the effectiveness of approaches.

For peer review only - http://bmjopen.bmj.com/site/about/guidelines.xhtml

Strengths and limitations of this study

- The systematic inclusion of contraceptive stigma in the search exposed a gap in the evidence of interventions designed to address this reproduction health stigma.
  The search strategy developed was designed to capture a broad swath of the literature for review.
- The interventions were organized according to an existing framework for abortion stigma interventions
- We limited our search to studies that were published in English which may have excluded key articles written in other languages.
- We focused on studies that had an element of evaluation, thereby excluding descriptions of activities taking place to reduce contraception and abortion stigma without efforts to measure effectiveness, acceptability or implementation.

For peer review only - http://bmjopen.bmj.com/site/about/guidelines.xhtml

Introduction

Health-related stigma has important implications for medical, social, and psychological wellbeing of those affected [1, 2, 3]. In reproductive health, stigmatizing attitudes towards people receiving or providing care is mediated by perceived alignment of practices surrounding human reproduction with societal morals and expectations [4, 5]. Contraception and abortion stigma have a range of health and non-health related consequences, such as unintended pregnancy, perceptions of promiscuity, criminalization, secrecy, unsafe abortion and associated complications, and poor quality of abortion care [6, 7]. Contraceptive use stigma emerges where there is censure of the control of fertility, and where contraceptive use conflicts with social norms regarding the life course timing and role of sexual activity, and desired family size [7, 8, 9]. Although rates vary by country [10], abortion is a routine component of sexual and reproductive health. Abortion stigma creates challenges and barriers to access to safe services that can result in deleterious physical, economic and social consequences for women [11, 12]. The two phenomenon are rooted in the negation of the right of inidviudals to decide whether and when to have children, make decisions about and have control of their body and sexuality, and manifest similarly in social norms and practices that hinder access to family planning and lead to unintended pregnancy [7].

Abortion stigma has been defined as, "…a negative attribute ascribed to women who seek to terminate a pregnancy that marks them, internally or externally, as inferior to the ideals of womanhood"[13]. Abortion stigma is located and manifests in various aspects of society and

For peer review only - http://bmjopen.bmj.com/site/about/guidelines.xhtml

affects a wide range of groups [14]. Kumar and colleagues [13] adapted a framework [15] to illustrate the levels of society at which abortion stigma operates and where interventions are needed. At the governmental/structural level abortion stigma is thought to operate in educational, economic, legal, and health policies that reflects social norms discrediting abortion. Internal procedures and guidelines that perpetuate abortion stigma characterize the institutional level. At the community level, abortion stigma influences social factors, attitudes and actions that cause individuals involved with abortion to lose status and social networks. Individual-level stigma, or the internalization of negative feelings, such as shame and guilt associated with the experience of abortion, manifests as intrapersonal and interpersonal stigma [13]. Investigation of contraceptive stigma is limited; however, one analysis with adolscents in Kenya determined that negative sterotypes and discrimination results in delays in access to health care and missed opportunities for contraceptive counselling [7, 16].

Eliminating stigma in sexual and reproductive health care is viewed as critical to avoid negative outcomes and for overall public health [17]. A recent scoping review explored the ways in which abortion stigma influences quality in abortion care [18] and determined that stigma reducing interventions are important for improving quality and decreasing unsafe abortion. A systematic review of multi-level health stigma reduction interventions concluded that research should expand the groups targeted [19]. Although, interventions to reduce contraception and abortion stigma exist [20], the volume, approaches employed, levels targeted, and effectiveness of these remain largely unknown.

For peer review only - http://bmjopen.bmj.com/site/about/guidelines.xhtml

We undertook a scoping review to identify the key characteristics of this evidence base and analyze gaps. A scoping review approach is appropriate for such indications [21, 22] and is a first step to be followed by assessments of effectiveness in systematic review. The aim of this scoping review was to identify the types, volume and characteristics of available evidence and analyze gaps in the knowledge base for interventions to reduce contraception and abortion stigma.

Methods

This review was conducted in accordance with Arskey and O'Malley's scoping review methodology and Levac and colleagues' methodological enhancement [23, 24]. We report the methodology and findings according to PRISMA (Preferred Reporting Items for Systematic Reviews and Meta-Analyses) guidelines [25] (Appendix 1).

Search terms

To locate literature for this scoping review, we developed a search strategy with support from experienced librarians in the World Health Organization (WHO) library (Appendix 2). We conducted this search in five electronic databases: MEDLINE, PubMED, EMBASE, Web of Science and PsychINFO to identify articles published between January 2000 and January 2022. We used the Covidence tool (Covidence Systematic Review Software, Veritas Health Innovation, Melbourne, Australia, available at www.covidence.org) to screen, review, organize and extract information from the articles.

For peer review only - http://bmjopen.bmj.com/site/about/guidelines.xhtml

Inclusion criteria

We used following eligibility criteria for inclusion in the scoping review: (1) published between January 2000 and January 2022, (2) written in English, (3) reports on the evaluation of an intervention specifically designed to reduce contraceptive and/or abortion stigma in any relevant population as part of the primary aim of the study, (4) uses any type of study design, and (5) was conducted in any country context.

Exclusion criteria

We excluded titles and abstracts if they were a review of other research [22] , correction, commentary, editorial, letter, case report, or guideline. Furthermore, we excluded articles that were unavailable through institutional holdings.

Data extraction

Two researchers independently screened the titles and abstracts against the inclusion and exclusion criteria. The full texts deemed relevant by both reviewers were retrieved and reviewed. Two researchers independently assessed the full-text articles to confirm eligibility and reconcile any discrepancies. We additionally conducted a search for grey literature of evaluations of interventions to reduce contraceptive and abortion stigma by exploring the websites of key

For peer review only - http://bmjopen.bmj.com/site/about/guidelines.xhtml

organizations working in sexual and reproductive health and databases designed to extract unpublished literature, but located no relevant texts during this process.

Data charting process and analysis

We developed a data-charting form to extract relevant information to answer our scoping review question. One researcher classified and charted all studies according to study location, study aim, study design, type of contraceptive method(s), study population, type of stigma, and intervention approach (Supplementary Table 1). The second researcher reviewed to 20 percent of extractions of included articles to identify any discrepancies. Both researchers met to discuss, re-review articles and resolve any conflicts.

For the analysis of the included literature, we drew upon an existing stigma intervention framework outlined in a 2013 White Paper [20, 26] including the following categories: counselling approaches, education and training (information and skills-building), social marketing, protest and contact with affected groups. Although designed to address abortion stigma, the framework drew upon evidence from a range of health-stigmas and thus may also apply to contraceptive stigma. The interventions included in the four categories aim to address intrapersonal and interpersonal stigma (Table 1). The White Paper included an additional three categories for interventions designed to address stigma at the structural level: institutions, laws and policies, and the media [20]. We used this overall framework to organize the literature included in this scoping review. Finally, study quality was not assessed, as this is not a requirement for scoping reviews [22, 23].

For peer review only - http://bmjopen.bmj.com/site/about/guidelines.xhtml

Table 1. Categories of analysis

Category	Definition
Type of stigma [13, 15]	
Intrapersonal	Internalization of negative feelings, such as shame and guilt, associated with experience with abortion
Interpersonal	Direct or enacted forms of stigma, such as verbal harassment and/or physical assault based on association with abortion
Community	Social factors and attitudes and actions that cause individuals involved with abortion to lose status in their communities and social networks
Organizational/Institutional	Procedures and guidelines internal to organizations that perpetuate abortion stigma
Governmental/Structural	Educational, economic, legal, health policies and other regulation that reflects social norms that discredit abortion
Intervention approach [20]	
Counselling/peer support	Provide healthcare information, emotional support, and empowerment to people who experience stigma
Education/training/skills building	Provision of information about the stigmatized group and their concerns with the goal of reducing stigma
Contact	In-person interactions that can lead to reductions in prejudice between groups
Protest	An action that publicly calls attention to stigmatizing attitudes and/or behaviours that promote these attitudes

For peer review only - http://bmjopen.bmj.com/site/about/guidelines.xhtml

Social marketing	A strategy that uses marketing techniques to achieve behavioural and/or health-related goals
Media	Extended forms of contact that may involve fictional and nonfictional media or visualizations

For peer review only

For peer review only - http://bmjopen.bmj.com/site/about/guidelines.xhtml

Patient and public involvement

We did not engage with patients or the public during the development of this review.

Findings

Our initial search resulted in 16,096 records. After removing 4,192 duplicate articles, two researchers independently screened 11,904 titles and abstracts against the inclusion and exclusion criteria (Figure 1). The full texts of the 196 titles and abstracts were retrieved and reviewed. Of these, 178 were excluded (Figure 1). The remaining 18 texts were included in this review (Figure 1).

Characteristics of included articles

The 18 included studies were conducted in 10 countries: Cambodia (1), Colombia (1), India (1), Indonesia (1), Kenya (1), Mexico [1] (3), South Africa (1), Spain (1), and the United States of America (USA)(8). One study included unspecified countries in the Latin America and Caribbean region and Sub-Saharan Africa [27]. Of the 18 included studies, 11 reported on a quantitative evaluation, 6 reported on a qualitative evaluation, and 1 used mixed methods approaches. Fourteen of the studies focussed exclusively on abortion stigma, and two studies focussed on

[1] One study was conducted in Mexico City.

For peer review only - http://bmjopen.bmj.com/site/about/guidelines.xhtml

contraception stigma. The remaining two considered both. A majority aimed to address intrapersonal stigma. We found no interventions designed to address stigma at the structural level. Table 1 presents the full list of included studies.

Description of stigma reduction interventions

Of the 18 included papers, seven were categorized as education/training/skills building, five were categorized as counselling/peer support, three as contact, and three as media (Table 1). Each intervention is described by category below.

Education/training/skills building

Values clarification is a training intervention for addressing thoughts, feelings and attitudes towards issues that are emotive, such as contraception and abortion. The first article, published in 2015, was an evaluation of the effectiveness of the *Difficult Dialogues* strategies in reducing negative attitudes among midwives in South Africa [28]. The qualitative study suggested that the intervention had some influence on increasing participants' empathy for their patients [28]. The second article evaluated the TEACH (Teaching Everything About Contraceptive Health) program which aimed to dispel myths about contraception and abortion among obstetrican-gynocology residents in a faith-based hospital in the USA. The results of the evaluation suggested that the intervention had limited impact on reducing discriminatory attitudes towards patients seeking abortion services [29]. Finally, a recent evaluation of another values clarification workshop

For peer review only - http://bmjopen.bmj.com/site/about/guidelines.xhtml

conducted with residents in Catholic hopsitals in the USA found that, when presented with abortion related-scenarios, residents held less stigmatizing attitudes [30].

Researchers in Spain implemented an intervention for nursing students including a simulation of family planning counselling session. Using a quasi-experimental design, the evaluation determined that two weeks after the intervention, students' attitudes about contraceptive methods improved as measured by relevant validated scales [31].

An evaluation of an intervention conducted in India aimed to empower women to seek services and to normalize abortion and the provision of support by the community and family members of women who seek services. Via a quasi-experimental approach, a scale was used to assess attitudes towards abortion following the intervention. The results suggested that it increased perceived levels of social support for abortion within their families and self-efficacy around family planning and abortion. However, perceptions of support for abortion within communities were lower after the intervention [32].

The Family Planning Balance Counselling Strategy (FP-BCS) is an intervention designed to support the uptake of modern contraceptive methods. Researchers evaluated the effectiveness of the FP-BCS on attitudes, subjective norms and intentions to use modern contraception in Indonesia. An evaluation using a quasi-experimental design determined a significant increase in positive attitudes towards modern contraception among mothers as a result [33].

For peer review only - http://bmjopen.bmj.com/site/about/guidelines.xhtml

An evaluation of a school-based intervention assessed whether Comprehensive Sexuality Education reduced stigma related to abortion and contraceptive use in western Kenya. Among students ages 13-21 at follow-up, stigma scores for both contraception and abortion reduced significantly among boys and girls [7].

Researchers developed a narrative intervention that promoted cognitive restructuring and was designed to normalize the abortion experience using a animated 4-minute short-film. People seeking abortion viewed the animation in the waiting room before receiving care. A randomized controlled trial assessed changes to internalized stigma as a result of the intervention. At two-week follow-up there was no significant difference internalized stigma between intervention and control groups [34].

Counselling/peer support

The Story Circles Project intervention was implemented in Mexico and designed to reduce intrapersonal stigma among women who had experienced an abortion. The Story Circles Project provided a safe space for women to share their abortion stories, connect with other women and explore their abortion decision. The intervention also included information sessions to dispel myths and misinformation. The authors found that the intervention significantly reduced women's feelings of isolation helped reduce stigma [35].

Three evaluations of the Provider Share Workshop, an intervention designed for health workers involved in abortion care, were included in this review [36, 37, 38]. The workshop sessions offer

For peer review only - http://bmjopen.bmj.com/site/about/guidelines.xhtml

providers an opportunity to discuss and explore their experiences. The 2011 pilot study found that participants reported an “increased interpersonal connection” with the team following the workshop [36]. In 2014, the Provider Share Workshop was conducted among providers in the USA and effectiveness was evaluated using two validated scales. Although the evaluation could not show statistically significant results, the study identified a downward trend in abortion stigma overtime [37]. Finally, the Workshop was adapted and piloted in two countries in Sub-Saharan Africa and Latin America. The evaluation found decreases in overall abortion stigma, but specifically in disclosure management, internalized stigma and judgement [38].

A qualitative assessment of the role of counselling within legal abortion services offered in non-governmental organizations in Mexico City and Colombia in addressing women’s concerns and fears prior to receiving abortion care found that women experienced relief, and less guilt and internalised stigma after counselling [38].

Contact

In 2018, researchers assessed the effectiveness of an intervention designed to reduce interpersonal abortion stigma manifesting as secrecy among women who have experienced abortion and negative reactions from women who learn about the abortion experiences of others. The intervention used the safe spaces of exisiting book clubs to assess whether women would self-disclose their abortion experiences in a positive face-to-face interaction. Book clubs were recruited to read and discuss the book *Choice: True Stories of Birth, Contraception, Infertility, Adoption, Single Parenthood and Abortion*. During the book club intervention, a majority of women

For peer review only - http://bmjopen.bmj.com/site/about/guidelines.xhtml

with abortion histories disclosed their experience and the book "warmed" participants feelings about abortion over time [39].

The online platform, Lightbulbs (*Focos*, in Spanish), was designed in Mexico to provide a safe space for women to tell their abortion stories and to increase abortion visibility. In discourse analysis of the text, researchers found that most of the stories were positive and described as a "cathartic" experience [40].

Media

In 2016, researchers examined in the impact of the viewing of the documentary film, *After Tiller*, in reducing third trimester abortion stigma. Viewing the film appeared to challenge myths and misperceptions about abortion. However, the authors found that the medium was limited in its ability to generate new belief systems around abortion [41].

The results of an online study of viewers on an episode of the USA medical television program *Grey's Anatomy* which featured an abortion storyline titled "Papa Don't Preach". The findings suggested that the program did not increase viewers willingness to support a friend seeking an abortion, whether medically necessary or induced [42].

Discussion

For peer review only - http://bmjopen.bmj.com/site/about/guidelines.xhtml

Despite calls for the development of interventions focussed on reducing reproductive health stigmas, and specifically, contraceptive and abortion stigma, there remains significant gaps in the literature [4, 16, 18, 19]. Most articles identified were reports of evaluations of abortion stigma interventions taking place in the USA. A handful of articles focussed on Latin America, two in Sub-Saharan Africa, one in Asia and one in Europe demonstrating limitations of understanding of available interventions in specific regions and contexts.

The published interventions largely focussed on addressing intrapersonal and interpersonal stigma. The most common intervention approach identified was education, training and skills building. The least common was the contact approach. There is a significant gap in the availability of interventions to reduce contraceptive and abortion stigma at the structural level and using approaches involving institutions, and laws and policies.

There exists substantial theory about intervention approaches that should have an effect on reducing contraception and abortion stigma [1, 15, 17, 18]. Several rigoursly validated scales are available for measuring changes in contraception and abortion stigma. Program developers and evaluators should design and test interventions based on theory and measure their effectiveness using existing validated scales. Future research may synthesize the existing evidence to understand the effectiveness of specific intervention approaches.

The methodology of this scoping review has some limitations. We limited our search to studies that were published in English; this may have inadvertently excluded key articles written in other

For peer review only - http://bmjopen.bmj.com/site/about/guidelines.xhtml

languages. We focused on studies that had an element of evaluation. This meant that we excluded descriptions of activities taking place to reduce contraception and abortion stigma without efforts to measure effectiveness, acceptability or implementation. This methodological choice may have limited our engagement with interventions currently in use, but not evaluated. It is also possible that evaluators have not published the results of studies of ineffective or inconclusive interventions.

In the design of interventions, stigma continues to be largely conceptualised as an attribute and interventions to mitigate stigma targeted to manifestations at the individual level. Any interventions aligning with this concept may inadvertently (re)generate stigma and minimize the impact of mitgation efforts. Further attention in the development of interventions targeted to the social processes that underpin stigma may have a more profound impact on attitudes and behaviours across populations [43]. Furthermore, efforts to reduce contraceptive and abortion stigma could draw upon a broader stigma framework and benefit from shared concepts, tools, indicators and outcomes [44]. Scoping reviews are pragamatic precursors to systematic reviews where the scope and characteristics of an area of investigation is unclear [22]. This results of this scoping review can inform efforts to develop robust systematic reviews to assess the quality and effectiveness of approaches to reduce contraceptive and abortion stigma.

Acknowledgements

The authors would like to acknowledge the input of Kavita Kothari, World Health Organization Librarian, Geneva, Switzerland for her suggestions and support in refining the search strategy.

For peer review only

Disclaimer

The views expressed in this article are those of the authors and do not necessarily represent the views of, and should not be attributed to, the World Health Organization.

Funding

The UNDP-UNFPA-UNICEF-WHO-World Bank Special Programme of Research, Development and Research Training in Human Reproduction (HRP), a co-sponsored program executed by the World Health Organization (WHO), funded this work.

Competing interests None declared.

Patient consent for publication Not required

Provenance and peer review Not commissioned; externally peer reviewed.

Data availability statement All data relevant to the study are included in the article or uploaded as supplementary information.

Ethics approval An ethics statement is not applicable because this study was based exclusively on published literature.

For peer review only

Contributorship statement AMS and URL conceptualized and conducted the review, including assessing literature and data synthesis. AMS prepared the manuscript with intellectual contributions from URL. Both authors approved the final manuscript.

For peer review only

References

1. Scrambler G, Heijnders M and van Brakel WH on behalf of ICRAAS. (2006) Understanding and tackling health-related stigma. Psychology, Health & Medicine, 11:3, 269-270, DOI: 10.1080/13548500600594908

2. Hatzenbuehler ML, Phelan JC and Link BG. (2013) Stigma as a fundamental cause of population health inequalities. American Journal of Public Health, 103(5): 813-21

3. Ohlsson-Nevo E and Karlsson J. (2018) Impact of health-related stigma on psychosocial functioning in the general population: Construct validity and Swedish reference data for the Stigma-related Social Problems scale (SSP). Research in Nursing and Health, 42: 72-82.

4. Cook RJ and Dicken BM (2014) Reducing stigma in reproductive health. International Journal of Gynecology and Obstetrics, 125: 89-92.

5. Makenzius M, McKinney G, Oguttu M. et al. Stigma related to contraceptive use and abortion in Kenya: scale development and validation. Reprod Health 16, 136 (2019). https://doi.org/10.1186/s12978-019-0799-1

6. Ostrach B (2012) The tangled web of reproductive morbidity risk: Abortion stigma, safety and legality. Frontiers in Women's Health 1 (2)

7. Rehnström Loi U. (2020) Abortion, contraception and associated social stigma – consequences and solutions in a low-resource setting in Western Kenya. PhD Thesis. Karolinska Institutet, Stockholm, Sweden.

8. Blodgett M, Weidert K, Nieto-Andrade B, and Prata N. (2018) Do perceived contraception attitudes influence abortion stigma? Evidence from Luanda, Angola. Social Science and Medicine - Population Health; 5: 38-47.

For peer review only

9. Farmer DB, Berman L, Ryan G, Habumugisha L, Basinga P, Nutt C, Kamali F, Ngizwenayo E, St. Fluer J, Niyigena P, Ngabo F, Farmer PE and Rich ML. (2015) Motivations and contraints to family planning: A qualitative study in Rwanda's Southern Kayonza District. Global Health: Science and Practice, 3(2):242-254; https://doi.org/10.9745/GHSP-D-14-00198

10. Bearak JM, Popinchalk A, Beavin C, Ganatra B, Moller AB, Tunçalp Ö, and Alkema L. (2022) Country-specific estimates of unintended pregnancy and abortion incidence: a global comparative analysis of levels in 2015–2019. BMJ Global Health;7:e007151. doi:10.1136/ bmjgh-2021-007151

11. Hanschmidt F, Linde K, Hilbert A, Riedel-Heller SG and Kersting A (2016) Abortion stigma: A systematic review. Perspectives on Sexual and Reproductive Health, 48(4): doi: 10.1363/48e8516

12. Moore B, Poss C, Coast E, Lattof SR, van der Meulen Rodgers Y (2021) The economics of abortion and its links with stigma: A secondary analysis from a scoping review on the economics of abortion. PLoS ONE 16(2): e0246238. https://doi.org/10.1371/journal.pone.0246238

13. Kumar A, Hesseni L and Mitchell EMH (2009) Conceptualising abortion stigma. Culture, Health and Sexuality, Aug;11(6):625-39. doi: 10.1080/13691050902842741.

14. Norris A, Bessett D, Steinberg JR, Kavanaugh ML, De Zordo S, and Becker D. (2011) Abortion stigma: A reconceptualization of constituents, causes, and consequences. Women's Health Issues, doi:10.1016/j.whi.2011.02.010

15. Heijinders M and van Der Meij (2006) The fight against stigma: an overview of stigma-reduction strategies and interventions. Psychol Health Med, Aug;11(3):353-63. doi: 10.1080/13548500600595327

For peer review only - http://bmjopen.bmj.com/site/about/guidelines.xhtml

16. Rehnström Loi U, Otieno B, Oguttu M, Gemzell-Danielsson K, Klingberg-Allvin M, Faxelid E and Makenzius M (2019) Abortion and contraceptive use stigma: a cross-sectional study of attitudes and beliefs in secondary school students in western Kenya. Sexual and Reproductive Health Matters, 27:3, 20-31, DOI: 10.1080/26410397.2019.1652028

17. Hussein J and Ferguson L (2019) Eliminating stigma and discrimination in sexual and reproductive health care: a public health imperative, Sexual and Reproductive Health Matters, 27:3, 1-5, DOI: 10.1080/26410397.2019.1697103

18. Sorhaindo A and Lavelanet AF. Why does abortion stigma matter? A scoping review and conceptual framework describing the role of stigma in shaping abortion quality of care. *Social Science and Medicine*, https://doi.org/10.1016/j.socscimed.2022.115271.

19. Rao D, Elshafei A, Nguyen M, Hatzenbuehler ML, Frey S and Go VF. (2019) A systematic review of multi-level stigma interventions: state of the science and future directions. 17:41 https://doi.org/10.1186/s12916-018-1244-y

20. Cockrill K, Herold S, Blanchard K, Grossman D, Upadhyay U, Baum S. (2013a). Addressing Abortion Stigma Through Service Delivery: A White Paper. Retrieved from Ibis Reproductive Health: https://www.ibisreproductivehealth.org/publications/addressing-abortion-stigma-through-service-delivery-white-paper.

21. Armstrong R, Hall BJ Doyle J and Waters E. (2011) “Scoping the scope” of a cochrane review. Journal of Public Health, 33(1): 147-150.

22. Munn Z, Peters MDJ, Stern C, Tufanaru, McArthur A and Aromataris E. (2018) Systematic review or scoping review? Guidance for authors when choosing between a systematic or scoping review approach. BMC Medical Research Methodology, 18:143, https://doi.org/10.1186/s12874-018-0611-x

For peer review only - http://bmjopen.bmj.com/site/about/guidelines.xhtml

23. Arksey, H and O'Malley, L (2005) Scoping studies: towards a methodological framework. International Journal of Social Research Methodology, 19-32.

24. Levac D, Colquhoun H, O'Brien KK. (2010) Scoping studies: advancing the methodology. Implement Sci, 5:69.

25. Moher D, Shamseer L, Clarke M, Ghersi D, Liberati A, Petticrew M et al. (2015) Preferred reporting items for systematic review and meta-analysis protocols (PRISMA-P) 2015 statement. Syst Rev, 4: 1 https://doi.org/10.1186/2046-4053-4-1

26. Brown, L., Macintyre, K., & Trujillo, L. (2003). Interventions to reduce HIV/AIDS stigma: What have we learned? AIDS Education and Prevention, 15(1), 49-69.

27. Mosley EA, Martin L, Seewald M, Hassinger J, Blanchard K, Baum SE, Santana D, Echeverri L, Garrett J, Njunguru J and Harris L (2020). Addressing abortion provider stigma: A pilot implementation of the Providers Share Workshop in sub-Saharan Africa and Latin America. International Perspectives on Sexual and Reproductive Health, 46: 35-50.

28. Mpeli MR and Botma Y (2015) Abortion-related services: Values clarification through ‘Diffcult Dialogues’ strategies. Education, Citizenship and Social Justice, 10(3): 278-288

29. Guiahi M, Cortland C, Graham MJ, Heraty S, Lukens M, Trester M, Summers S, Kenton K (2011) Addressing OB/GYN family planning educational objectives at a faith-based institution using the TEACH program. Contraception, 83: 367-372.

30. Guiahi M, Wilson C, Claymore E, Simonson K, Steinauer J. (2021) Influence of a values clarification workshop on residents training at Catholic Hospital programs. Contraception: X 3 100054

31. Sanz-Martos S, López-Medina IM, Álvarez-Garcia C, Álvarez-Nieto C. (2021) Educational program on sexuality and contraceptive methods in nursing degree students. Nurse Education Today, 107: 105114

For peer review only - http://bmjopen.bmj.com/site/about/guidelines.xhtml

32. Banerjee SK, Andersen KL, Warvadekar J and Pearson E. (2013) Intervention to improve knowledge and perceptions abortion abortion in Bihar and Jharkhand, India. International Perspectives in Sexual and Reproductive Health, 39(3): 142-151, doi:10.1363/3914213

33. Palinggi RS, Moedjiono AI, Suarayasa K, Masni, Seweg A, Amqam H, Nur R amd Syam A (2021) The effect of balanced counseling strategy family planning against attitude, subjective norm, and intentions on the use of modern contraception behavior in the Singgani Public Health Center work area of Palu city. Gac Sanit

34. Sackeim MG, Lee SY, Newton S, Lauderdale D, Gilliam M (2022). Effect of a narrative intervention on individual-level abortion stigma: a randomised controlled trial. BMJ Sexual and Reproductive Health; 48:47–53.

35. Belfrage M, Ortíz Ramírez O and Sorhaindo A. (2019) Story Circles and abortion stigma in Mexico: a mixed-methods evaluation of a new intervention for reducing individual level abortion stigma, Culture, Health & Sexuality, DOI: 10.1080/13691058.2019.1577493

36. Harris LH, Debbink M, Martin L, and Hassinger J (2011) Dynamics of stigma in abortion work: Findings from a pilot of the Providers Share Workshop. Social Science and Medicine, 73: 1062-1070.

37. Martin LA, Debbink M, Hassinger J, Youatt E and Harris LH. (2014) Abortion providers, stigma and professional quality of life. Contraception, 90: 581-587.

38. Keefe-Oates B, Makleff S, Sa E, Forero LJ, Mendoza D, Olaya MA. (2019) Experiences with abortion counselling in Mexico City and Colombia: addressing women's fears and concerns. Culture, Health and Sexuality, 4.

39. Cockrill K and Biggs A. (2018) Can stories reduce abortion stigma? Findings from a longitudinal cohort study. Culture, Health and Sexuality, 3.

For peer review only - http://bmjopen.bmj.com/site/about/guidelines.xhtml

40. Belfrage M, Didier E and Vázquez-Quesada L (2021): Voicing Abortion Experiences to Reduce Stigma: Lessons from an Online Storytelling Platform in Mexico, Women's Reproductive Health, DOI: 10.1080/23293691.2021.2016145

41. Sisson G and Kimport K (2016) After After Tiller: the impact of a documentary film on understandings of third-trimester abortion. Culture, Health and Sexuality: 6.

42. Sisson G, Walter N, Harold S and Brooks JJ. (2021) Prime-time abortion on Grey's Anatomy: What do US viewers learn from fictional portrayals of abortion on television?

43. Millar E. (2020) Abortion stigma is a social process. Women's Studies International Forum, 78: 102328

44. Stangl AL, Earnshaw VA, Logie CH, van Brakel W, Simbayi LC, Barré I and Dovidio JF. (2019) The Health Stigma and Discrimination Framework: a global, crosscutting framework to inform research, intervention development, and policy on health-related stigmas. BMC Medicine, 17:31

For peer review only

Figure 1. Scoping review PRISMA flowchart

For peer review only

Figure 1. Scoping review PRISMA flowchart

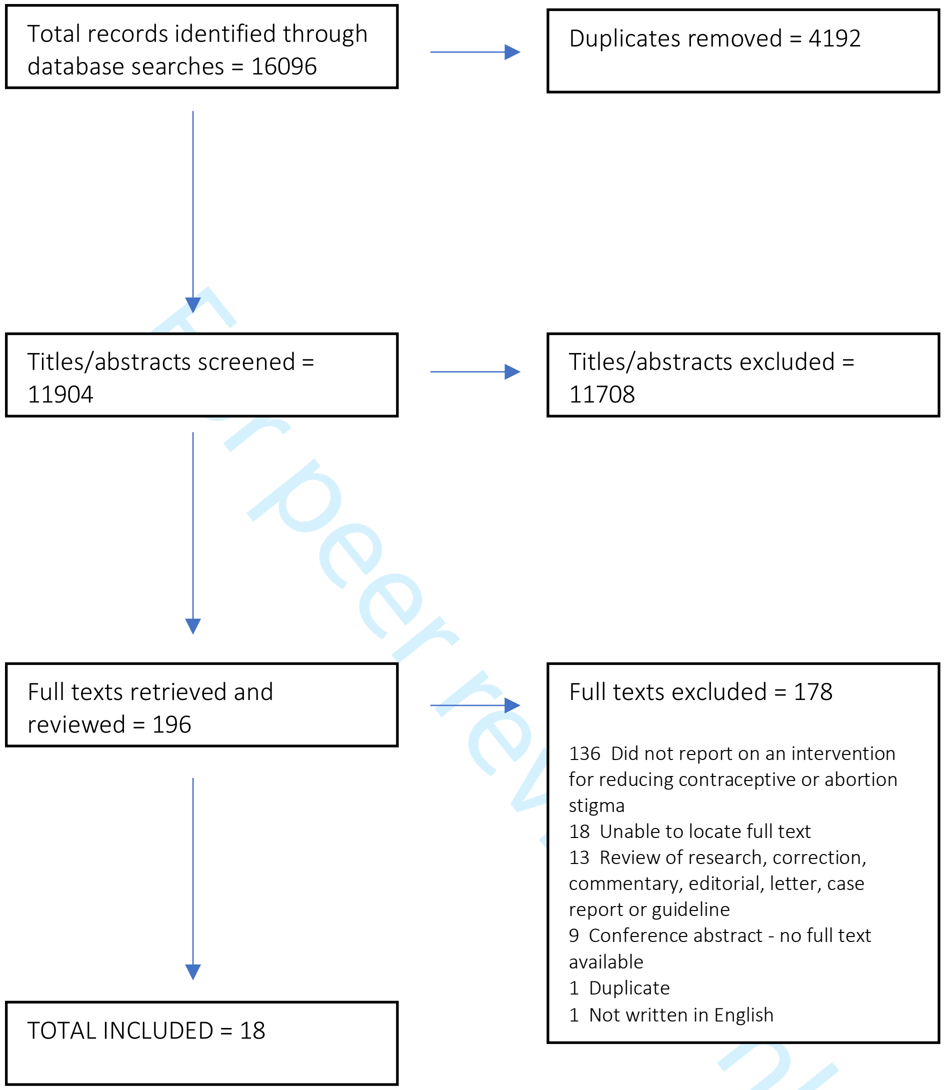

For peer review only

Supplemental material

Supplementary Table 1. Characteristics of included studies (n=18)

Author	Year of publication	Country	Scope	Evaluation design	Focus	Type of stigma	Intervention approach
Banerjee	2013	India	To evaluate the effectiveness of a behavior change communication intervention to improve women's knowiedge about India's abortion law and their perceptions about abortion	Quasi-experimental	Abortion	Community	Education/training/skills building
Belfarge	2020	Mexico	The evaluation measured whether participation in the intervention reduced women's experience of individual level stigma one month after having participated and explored women's expressed experiences of the intervention and their perceptions of changes in stigmatising feelings after the intervention.	Mixed methods	Abortion	Intrapersonal	Counselling/peer support
Belfarge	2021	Mexico	To understand more about who tells their stories online, how they represent their abortion experience, and what lessons this leaves us as feminist activists seeking to advance sexual and reproductive rights through online storytelling	Qualitative	Abortion	Intrapersonal	Contact
Cockrill	2018	USA	To measure changes in women's attitudes toward abortion and their willingness to disclose their own abortion experiences with one another	Quasi-experimental	Abortion	Interpersonal	Contact

For peer review only

			following a book group discussion in which participants discussed a previously chosen book.				
Guiahi	2011	USA	To evaluate if a values clarification workshop conducted at Catholic hospital training programs influenced obstetrics and gynecology residents' abortion attitudes.	Quasi-experimental	Abortion	Interpersonal	Education/training/skills building
Guiahi	2021	USA	To understand whether a values clarification workshop influences abortion attitudes of obgyn residents at U.S. training programs that are based in Catholic hospitals and lack abortion training.	Quasi-experimental	Abortion	Interpersonal	Education/training/skills building
Harris	2011	USA	To present findings from a qualitative pilot study of the Providers Share Workshop, a six-session workshop in which abortion providers (broadly defined to include anyone with direct daily involvement in abortion care), meet to explore their experiences, guided by an experienced facilitator	Qualitative	Abortion	Intrapersonal	Counselling/peer support
Keefe-Oates	2020	Colombia and Mexico City	To develop a greater understanding of their clients' concerns prior to abortion care,and the role that counselling can play in addressing these concerns.	Qualitative	Abortion	Intrapersonal	Counselling/peer support
Martin	2014	USA	The Providers Share Workshop (PSW) provides abortion providers safe space to discuss their work experiences. Our	Quasi-experimental	Abortion	Intrapersonal	Counselling/peer support

For peer review only

			objectives were to assess changes in abortion stigma over time and explore how stigma is related to aspects of professional quality of life, including compassion satisfaction, burnout and compassion fatigue for providers participating in the workshops.				
Mosley	2020	Sub-Saharan Africa and Latin American and the Caribbean*	To adapt the Providers Share Workshop content, structure and evaluation tools for this pilot study in Sub-Saharan Africa and Latin America.	Cohort	Abortion	Intrapersonal	Counselling/peer support
Mpeli	2015	South Africa	To report on the value clarification process and the views of preregistration midwifery students regarding abortion-related services before and after implementing the value clarification process.	Qualitative	Abortion	Intrapersonal	Education/training/skills building
Palinggi	2021	Indonesia	To analyze the influence of the Balanced Counseling Strategy (FP-BCS) on attitudes, subjective norms, and intentions on modern contraception in the working area of Singgani Health Center, Palu, Indonesia	Quasi-experimental	Contraception	Community	Education/training/skills building
Rehnström Loi	2020	Kenya	To measure attitudes towards abortion and contraceptive use, and to evaluate a stigma-reduction intervention among secondary school students.	Quasi-experimental	Both	Interpersonal	Education/training/skills building

For peer review only

Sackeim	2022	USA	To evaluate the effect of a narrative intervention on individual-level abortion stigma in patients undergoing abortion.	Randomized Controlled Trial	Abortion	Intrapersonal	Contact
San-Martos	2021	Spain	This study aimed to evaluate the effect of a peer-educational intervention to improve knowledge level and attitudes regarding contraceptive methods in university students.	Quasi-experimental	Contraception	Community	Education/training/skills building
Sisson	2016	USA	To determine whether a documentary film can impact stigmatizing attaitudes against 3rd trimester abortion.	Qualitative	Abortion	Community	Media
Sisson	2021	USA	To examine the impact of an abortion storyline from the TV show Grey's Anatomy on USA-based viewers.	Quasi-experimental	Abortion	Community	Media
Smith	2021	Cambodia	The aim of this study was to describe the development of an intervention to support the reproductive health of garment factory workers in Cambodia	Qualitative	Both	Community	Media

*Only regions were report

For peer review only

Appendix 1. Preferred Reporting Items for Systematic reviews and Meta-Analyses extension for Scoping Reviews (PRISMA-ScR) Checklist

SECTION	ITEM	PRISMA-ScR CHECKLIST ITEM	REPORTED ON PAGE #
**TITLE**			
Title	1	Identify the report as a scoping review.	1
**ABSTRACT**			
Structured summary	2	Provide a structured summary that includes (as applicable): background, objectives, eligibility criteria, sources of evidence, charting methods, results, and conclusions that relate to the review questions and objectives.	2
**INTRODUCTION**			
Rationale	3	Describe the rationale for the review in the context of what is already known. Explain why the review questions/objectives lend themselves to a scoping review approach.	4
Objectives	4	Provide an explicit statement of the questions and objectives being addressed with reference to their key elements (e.g., population or participants, concepts, and context) or other relevant key elements used to conceptualize the review questions and/or objectives.	4
**METHODS**			
Protocol and registration	5	Indicate whether a review protocol exists; state if and where it can be accessed (e.g., a Web address); and if available, provide registration information, including the registration number.	N/A
Eligibility criteria	6	Specify characteristics of the sources of evidence used as eligibility criteria (e.g., years considered, language, and publication status), and provide a rationale.	5
Information sources*	7	Describe all information sources in the search (e.g., databases with dates of coverage and contact with authors to identify additional sources), as well as the date the most recent search was executed.	5
Search	8	Present the full electronic search strategy for at least 1 database, including any limits used, such that it could be repeated.	26
Selection of sources of evidence†	9	State the process for selecting sources of evidence (i.e., screening and eligibility) included in the scoping review.	5
Data charting process‡	10	Describe the methods of charting data from the included sources of evidence (e.g., calibrated forms or forms that have been tested by the team before their use, and whether data charting was done independently or in	5-6

For peer review only

SECTION	ITEM	PRISMA-ScR CHECKLIST ITEM	REPORTED ON PAGE #
		duplicate) and any processes for obtaining and confirming data from investigators.	
Data items	11	List and define all variables for which data were sought and any assumptions and simplifications made.	22
Critical appraisal of individual sources of evidence§	12	If done, provide a rationale for conducting a critical appraisal of included sources of evidence; describe the methods used and how this information was used in any data synthesis (if appropriate).	N/A
Synthesis of results	13	Describe the methods of handling and summarizing the data that were charted.	6
RESULTS			
Selection of sources of evidence	14	Give numbers of sources of evidence screened, assessed for eligibility, and included in the review, with reasons for exclusions at each stage, ideally using a flow diagram.	15
Characteristics of sources of evidence	15	For each source of evidence, present characteristics for which data were charted and provide the citations.	16-21
Critical appraisal within sources of evidence	16	If done, present data on critical appraisal of included sources of evidence (see item 12).	N/A
Results of individual sources of evidence	17	For each included source of evidence, present the relevant data that were charted that relate to the review questions and objectives.	Click here to enter text.
Synthesis of results	18	Summarize and/or present the charting results as they relate to the review questions and objectives.	7-9
DISCUSSION			
Summary of evidence	19	Summarize the main results (including an overview of concepts, themes, and types of evidence available), link to the review questions and objectives, and consider the relevance to key groups.	9-10
Limitations	20	Discuss the limitations of the scoping review process.	10
Conclusions	21	Provide a general interpretation of the results with respect to the review questions and objectives, as well as potential implications and/or next steps.	9-10
FUNDING			
Funding	22	Describe sources of funding for the included sources of evidence, as well as sources of funding for the scoping review. Describe the role of the funders of the scoping review.	10

JBI = Joanna Briggs Institute; PRISMA-ScR = Preferred Reporting Items for Systematic reviews and Meta-Analyses extension for Scoping Reviews.

* Where *sources of evidence* (see second footnote) are compiled from, such as bibliographic databases, social media platforms, and Web sites.

† A more inclusive/heterogeneous term used to account for the different types of evidence or data sources (e.g., quantitative and/or qualitative research, expert opinion, and policy

For peer review only

documents) that may be eligible in a scoping review as opposed to only studies. This is not to be confused with *information sources* (see first footnote).
‡ The frameworks by Arksey and O'Malley (6) and Levac and colleagues (7) and the JBI guidance (4, 5) refer to the process of data extraction in a scoping review as data charting.
§ The process of systematically examining research evidence to assess its validity, results, and relevance before using it to inform a decision. This term is used for items 12 and 19 instead of "risk of bias" (which is more applicable to systematic reviews of interventions) to include and acknowledge the various sources of evidence that may be used in a scoping review (e.g., quantitative and/or qualitative research, expert opinion, and policy document).

From: Tricco AC, Lillie E, Zarin W, O'Brien KK, Colquhoun H, Levac D, et al. PRISMA Extension for Scoping Reviews (PRISMAScR): Checklist and Explanation. Ann Intern Med. 2018;169:467–473. doi: 10.7326/M18-0850.

For peer review only

Appendix 2. Example search strategy

	#	Searches
Stigma		Social stigma* OR shame* OR guilt* OR discriminat* OR Prejudice* OR judgement OR stereotype*OR blame OR isolate* OR moral* OR harass* OR insult* OR labeling OR social-accept* OR social-approv* OR social-percep*OR treatment-barrier*
Abortion or contraception		abortion, induced OR Abortion, Criminal OR Abortion, Septic OR Abortion Applicants OR abortion, missed OR abortion, legal OR abortion, spontaneous OR abortion* OR pregnancy-terminat* OR post-abortion*OR Postconception Fertility Control OR Embryotomies OR Embryotomy OR Dilatation and curettage OR Vacuum Curettage OR Surgical abortion OR Manual vacuum aspiration OR (vacuum AND aspiration) OR Curettage OR Surgical termination of pregnancy OR Dilatation and evacuation OR Dilation and evacuation OR Suction aspiration OR Aspiration abortion OR Suction curettage OR Vacuum curettage OR Contraception OR Contraception Behavior OR Condom OR Condoms OR Diaphragm OR Diaphragms OR Cervical Cap OR Vaginal-sponge OR Cervical-Caps OR Vaginal-sponges OR Birth-Control OR Contraceptive-Methods OR Contraceptive-Method OR Female Contraception OR Female Contraceptions OR Male Contraception OR Male Contraceptions OR Inhibition-of-Fertilization OR Fertilization-Inhibition OR Fertility-Control OR Birth-Control OR Vaginal-Barrier OR Hormonal-Contracept* OR anticonception OR antifertility OR conception-control OR family planning OR birth control OR birth-prevention OR pregnancy-prevent*
Program evaluation-related		intervention OR program OR activity OR action

For peer review only

Research Checklist

Appendix 1. Preferred Reporting Items for Systematic reviews and Meta-Analyses extension for Scoping Reviews (PRISMA-ScR) Checklist

SECTION	ITEM	PRISMA-ScR CHECKLIST ITEM	REPORTED ON PAGE #
**TITLE**			
Title	1	Identify the report as a scoping review.	1
**ABSTRACT**			
Structured summary	2	Provide a structured summary that includes (as applicable): background, objectives, eligibility criteria, sources of evidence, charting methods, results, and conclusions that relate to the review questions and objectives.	2
**INTRODUCTION**			
Rationale	3	Describe the rationale for the review in the context of what is already known. Explain why the review questions/objectives lend themselves to a scoping review approach.	4
Objectives	4	Provide an explicit statement of the questions and objectives being addressed with reference to their key elements (e.g., population or participants, concepts, and context) or other relevant key elements used to conceptualize the review questions and/or objectives.	4
**METHODS**			
Protocol and registration	5	Indicate whether a review protocol exists; state if and where it can be accessed (e.g., a Web address); and if available, provide registration information, including the registration number.	N/A

For peer review only

SECTION	ITEM	PRISMA-ScR CHECKLIST ITEM	REPORTED ON PAGE #
Eligibility criteria	6	Specify characteristics of the sources of evidence used as eligibility criteria (e.g., years considered, language, and publication status), and provide a rationale.	5
Information sources*	7	Describe all information sources in the search (e.g., databases with dates of coverage and contact with authors to identify additional sources), as well as the date the most recent search was executed.	5
Search	8	Present the full electronic search strategy for at least 1 database, including any limits used, such that it could be repeated.	26
Selection of sources of evidence†	9	State the process for selecting sources of evidence (i.e., screening and eligibility) included in the scoping review.	5
Data charting process‡	10	Describe the methods of charting data from the included sources of evidence (e.g., calibrated forms or forms that have been tested by the team before their use, and whether data charting was done independently or in duplicate) and any processes for obtaining and confirming data from investigators.	5-6
Data items	11	List and define all variables for which data were sought and any assumptions and simplifications made.	22
Critical appraisal of individual sources of evidence§	12	If done, provide a rationale for conducting a critical appraisal of included sources of evidence; describe the methods used and how this information was used in any data synthesis (if appropriate).	N/A
Synthesis of results	13	Describe the methods of handling and summarizing the data that were charted.	6

For peer review only

SECTION	ITEM	PRISMA-ScR CHECKLIST ITEM	REPORTED ON PAGE #
**RESULTS**			
Selection of sources of evidence	14	Give numbers of sources of evidence screened, assessed for eligibility, and included in the review, with reasons for exclusions at each stage, ideally using a flow diagram.	15
Characteristics of sources of evidence	15	For each source of evidence, present characteristics for which data were charted and provide the citations.	16-21
Critical appraisal within sources of evidence	16	If done, present data on critical appraisal of included sources of evidence (see item 12).	N/A
Results of individual sources of evidence	17	For each included source of evidence, present the relevant data that were charted that relate to the review questions and objectives.	Click here to enter text.
Synthesis of results	18	Summarize and/or present the charting results as they relate to the review questions and objectives.	7-9
**DISCUSSION**			
Summary of evidence	19	Summarize the main results (including an overview of concepts, themes, and types of evidence available), link to the review questions and objectives, and consider the relevance to key groups.	9-10
Limitations	20	Discuss the limitations of the scoping review process.	10
Conclusions	21	Provide a general interpretation of the results with respect to the review questions and objectives, as well as potential implications and/or next steps.	9-10
**FUNDING**			

For peer review only

SECTION	ITEM	PRISMA-ScR CHECKLIST ITEM	REPORTED ON PAGE #
Funding	22	Describe sources of funding for the included sources of evidence, as well as sources of funding for the scoping review. Describe the role of the funders of the scoping review.	10

JBI = Joanna Briggs Institute; PRISMA-ScR = Preferred Reporting Items for Systematic reviews and Meta-Analyses extension for Scoping Reviews.
* Where *sources of evidence* (see second footnote) are compiled from, such as bibliographic databases, social media platforms, and Web sites.
† A more inclusive/heterogeneous term used to account for the different types of evidence or data sources (e.g., quantitative and/or qualitative research, expert opinion, and policy documents) that may be eligible in a scoping review as opposed to only studies. This is not to be confused with *information sources* (see first footnote).
‡ The frameworks by Arksey and O'Malley (6) and Levac and colleagues (7) and the JBI guidance (4, 5) refer to the process of data extraction in a scoping review as data charting.
§ The process of systematically examining research evidence to assess its validity, results, and relevance before using it to inform a decision. This term is used for items 12 and 19 instead of "risk of bias" (which is more applicable to systematic reviews of interventions) to include and acknowledge the various sources of evidence that may be used in a scoping review (e.g., quantitative and/or qualitative research, expert opinion, and policy document).

From: Tricco AC, Lillie E, Zarin W, O'Brien KK, Colquhoun H, Levac D, et al. PRISMA Extension for Scoping Reviews (PRISMAScR): Checklist and Explanation. Ann Intern Med. 2018;169:467–473. doi: 10.7326/M18-0850.

For peer review only

For peer review only
